# Physiological intron retaining transcripts in the cytoplasm abound during human motor neurogenesis

**DOI:** 10.1101/gr.276898.122

**Published:** 2022-10

**Authors:** Marija Petrić Howe, Hamish Crerar, Jacob Neeves, Jasmine Harley, Giulia E. Tyzack, Pierre Klein, Andres Ramos, Rickie Patani, Raphaëlle Luisier

**Affiliations:** 1 The Francis Crick Institute, London NW1 1AT, United Kingdom;; 2 Department of Molecular Neuroscience, UCL Institute of Neurology, London WC1N 3AR, United Kingdom;; 3 Research Department of Structural and Molecular Biology, University College London, London WC1E 6XA, United Kingdom;; 4 Idiap Research Institute, Genomics and Health Informatics, CH-1920 Martigny, Switzerland;; 5 SIB Swiss Institute of Bioinformatics, 1015 Lausanne, Switzerland

## Abstract

Intron retention (IR) is now recognized as a dominant splicing event during motor neuron (MN) development; however, the role and regulation of intron-retaining transcripts (IRTs) localized to the cytoplasm remain particularly understudied. Here we show that IR is a physiological process that is spatiotemporally regulated during MN lineage restriction and that IRTs in the cytoplasm are detected in as many as 13% (*n* = 2297) of the genes expressed during this process. We identify a major class of cytoplasmic IRTs that are not associated with reduced expression of their own genes but instead show a high capacity for RNA-binding protein and miRNA occupancy. Finally, we show that ALS-causing *VCP* mutations lead to a selective increase in cytoplasmic abundance of this particular class of IRTs, which in turn temporally coincides with an increase in the nuclear expression level of predicted miRNA target genes. Altogether, our study identifies a previously unrecognized class of cytoplasmic intronic sequences with potential regulatory function beyond gene expression.

Intron retention (IR), a mode of alternative splicing (AS) whereby one or more introns are retained within mature polyadenylated mRNAs, has been greatly understudied in mammalian systems and for a long time was mostly considered as a product of inefficient or erroneous splicing. With advances in detection strategies, IR is now recognized as a more widespread and regulated process than previously thought, and the idea that IR could even functionally modulate cellular processes has come into focus, with its role(s) in cellular physiology beginning to unfold (Braunschweig et al. 2014; Boutz et al. 2015).

Neural cells have a higher proportion of transcripts with retained introns compared with other cell types, and an expanding body of evidence suggests a functional role for IR both in neuronal development and homeostasis (Buckley et al. 2011; Yap et al. 2012; Braunschweig et al. 2014; Mauger et al. 2016). Transcripts with IR are often detained in the nucleus as a means of reducing expression levels of transcripts not required for cellular physiology at that particular time (Xu et al. 2008; Boutz et al. 2015; Braun et al. 2017). Some of these transcripts will eventually be degraded by the nuclear exosome, whereas specific signals could stimulate splicing of the retained intron in others, resulting in export of the fully spliced mRNA into the cytoplasm and its subsequent translation (Mauger et al. 2016). Indeed, nuclear detention of intron-retaining transcripts (IRTs) provides a powerful mechanism to hold gene expression in a suppressed but poised state that allows rapid protein production if and when an appropriate stimulus is received (Ninomiya et al. 2011; Kalyna et al. 2012; Yap et al. 2012; Boothby et al. 2013; Mauger et al. 2016).

Although the stable cytoplasmic localization of intronic sequences in neurons has been reported since 2013 (Khaladkar et al. 2013), there has been limited investigation into the possible role of cytoplasmic IRTs. This has presumably been overlooked in part owing to detection limitations but also owing to a notion that these transcripts would likely contain premature translation termination codons (PTCs) and, as such, be degraded by nonsense-mediated mRNA decay (NMD) (Jaillon et al. 2008). Nevertheless, the NMD process is only partially efficient (Lindeboom et al. 2016; Dyle et al. 2020), and conditions exist in which a PTC does not lead to NMD (Lykke-Andersen and Jensen 2015). Although examples of IR coupled with NMD have been found to down-regulate gene expression, such as in granulocyte development (Wong et al. 2013), these transcripts can also encounter other fates in the cell (Vanichkina et al. 2018). Indeed, one of the few studies focusing on cytoplasmic IR in neurons showed an “addressing” function for intronic RNA sequences, determining the spatial localization of their host transcripts within cellular compartments such as dendrites (Sharangdhar et al. 2017). Another speculated function of IR has arisen following the finding that retained introns can be enriched in miRNA binding sites compared with nonretained introns (Schmitz et al. 2017). This offers an intriguing route through which miRNA-directed degradation pathways might regulate the abundance of IRTs; alternatively, the retained introns themselves may serve as miRNA sinks or even encode novel miRNAs termed “mirtrons” (Schmitz et al. 2017; Monteuuis et al. 2019). Altogether, despite advances in our understanding of IR in neuronal cells, much remains unanswered.

The importance of investigating the roles of IR has been further corroborated by studies that show its relevance across a diverse range of neurodegenerative diseases (Jangi et al. 2017; Luisier et al. 2018; Adusumalli et al. 2019; Wang et al. 2020). One such example is amyotrophic lateral sclerosis (ALS) (Luisier et al. 2018), a rapidly progressive and incurable disease that leads to selective degeneration of motor neurons (MNs). ALS is characterized by protein inclusions and axonal degeneration and is fundamentally associated with RNA processing defects. Indeed, ALS-causing mutations occur in numerous genes encoding crucial regulators of RNA processing, which are normally expressed ubiquitously throughout development. Despite the growing number of causative gene mutations being identified in ALS, the precise etiology remains unknown, and early molecular pathogenic events remain poorly understood. We previously found that aberrant IR is a widespread phenomenon in ALS (Luisier et al. 2018), which has been corroborated by subsequent studies (Wang et al. 2020; Hogan et al. 2021). Moreover, we went on to show aberrant cytoplasmic IR as a widespread molecular phenomenon in *VCP*-related ALS (Tyzack et al. 2021). We showed that ALS-related aberrant cytoplasmic IRTs are predicted to bind extensively to RNA-binding proteins (RBPs), including those that are mislocalized in ALS, and we proposed that a subset of cytoplasmic intronic sequences serve as “blueprints” for the hallmark protein mislocalization events in *VCP*-related ALS (Neumann et al. 2006; Tyzack et al. 2021). This raises an exciting possibility that intronic RNA sequences play additional significant roles beyond their recognized nuclear function. Nevertheless, the role and physiological relevance of cytoplasmic IR during neuronal development and disease still remain largely unresolved.

The objective of this study was twofold: (1) to deepen our understanding of the role(s) of cytoplasmic IR in normal cellular physiology by resolving the spatiotemporal dynamics of IRTs underlying distinct stages of MN lineage restriction and (2) to decipher whether specific classes of IRTs become dysregulated in the context of disease by systematically examining the influence of ALS-causing *VCP* mutations on this process. To achieve this, we sought to characterize the spatiotemporal dynamics of IRTs by reanalyzing RNA-seq data from nuclear and cytoplasmic fractions of human induced pluripotent stem cells (hiPSCs) undergoing motor neurogenesis.

## Results

### Thirteen percent of genes expressed during early human motor neurogenesis produce IRTs localized to the cytoplasm

**Figure 1.**
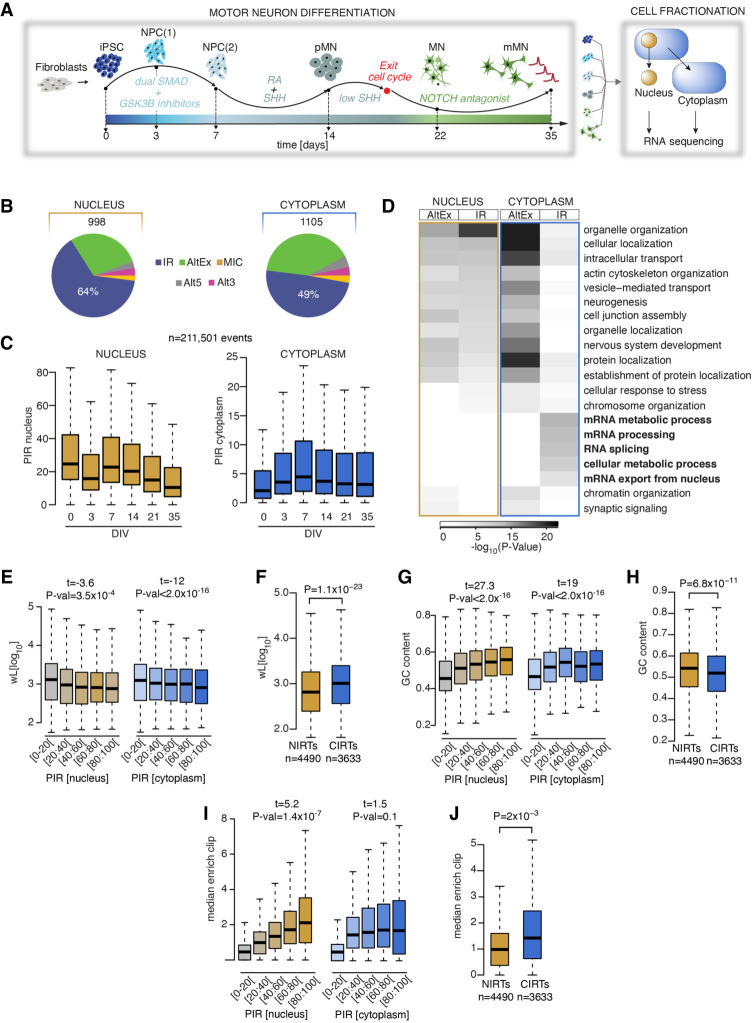
Nuclear and cytoplasmic intron retention (IR) affect two distinct mRNA subsets. (*A*) Schematic depicting the induced-pluripotent stem cell (iPSC) differentiation strategy for motor neurogenesis. Arrows indicate sampling time points in days when cells were fractionated into nuclear and cytoplasmic compartments before poly(A) RNA sequencing. Four iPSC lines were obtained from four independent healthy controls. (NPCs) Neural precursors, (pMNs) “patterned” precursor motor neurons (ventral spinal cord), (MNs) postmitotic but electrophysiologically inactive motor neurons, and (mMNs) electrophysiologically active MNs. (*B*) Pie charts representing proportions of pooled included splicing events in healthy control samples from distinct stages of motor neurogenesis compared with iPSCs or with a previous time point in nuclear (*left*) and cytoplasmic (*right*) fractions. Total number of events are indicated *above* the charts. (AltEx) Alternative exon, (MICs) microexons, and (Alt5 and Alt3) alternative 5′ and 3′ UTR. (*C*) Comparison of the percent intron retention (PIR) during MN differentiation in nucleus (*left*) and cytoplasm (*right*) for 21,161 events that show >10% PIR in at least three out of 47 nuclear samples. (*D*) Heatmap of the GO biological functions enriched among the genes showing AltEx or IR in either the nucleus or the cytoplasm. *P*-values obtained by Fisher's exact test. (*E*) Analysis of the relationship between intron length (wL) and the PIR in the nucleus (*left*) and the cytoplasm (*right*). *P*-values obtained from analysis of variance (see Methods). Retained introns are grouped in five categories of increasing level of retention, as indicated on the *x*-axis. (*F*) Comparison of intron length (wL) between nuclear and cytoplasmic retained introns. Nuclear retained introns are defined as introns showing >20% IR in the nuclear fraction and <5% IR in the cytoplasmic fraction. Cytoplasmic retained introns are defined as introns showing >20% IR in the nuclear fraction and >15% IR in the cytoplasmic fraction. *P*-values obtained from Mann–Whitney *U* test. (*G*) Analysis of the relationship between the PIR in the nucleus and %GC content. Data shown as in *E*. (*H*) Comparison of %GC content between nuclear and cytoplasmic retained introns. (*I*) Analysis of the relationship between the PIR in the nucleus and the median enrichment for RBP binding site, compared with the nonretained introns of the same gene. Data shown as in *E*. (*J*) Comparison of median enrichment for RBP binding sites between nuclear and cytoplasmic retained introns. (*C*,*E*–*J*) Data shown as box plots in which the *center* line is the median; limits are the interquartile range; and whiskers are the minimum and maximum.

We previously reported a transient IR program during early human motor neurogenesis using whole-cell RNA sequencing data (Luisier et al. 2018). To further examine the spatiotemporal dynamics of IR during this process in healthy cells, we reanalyzed high-throughput poly(A) RNA-seq data derived from nuclear and cytoplasmic fractions of hiPSCs (day 0), neural precursors (NPCs; day 3 and day 7), “patterned” ventral spinal motor neuron precursors (pMNs; day 14), postmitotic but electrophysiologically immature MNs (day 22), and electrophysiologically active MNs (mMNs; day 35; 23 nuclear and 24 cytoplasmic samples from six time points, four clones from four different healthy controls; one nuclear iPSC sample was removed owing to low quality) (Fig. 1A; Supplemental Table S1; Hall et al. 2017; Tyzack et al. 2021). Using the RNA-seq pipeline VAST-TOOLS (Irimia et al. 2014), we first identified 4189 nuclear and 1542 cytoplasmic significant (included or skipped) AS events between the five stages of motor neurogenesis (Supplemental Fig. S1A,B). In line with our previous study (Luisier et al. 2018), IR was the predominant mode of AS during neurodevelopment, accounting for 64% and 49% of the included AS events in the nucleus (638 events) and the cytoplasm (541 events), respectively (Fig. 1B). Further examination of the distributions of percent intron retention (PIR) during MN differentiation in the nucleus and the cytoplasm for 211,501 introns revealed that IR shows distinct dynamics in the two compartments (Fig. 1C). In particular, the nuclear compartment shows the highest level of PIR at the hiPSC stage, whereas the cytoplasmic compartment shows the highest level of PIR at DIV = 7, which is reminiscent of the early wave of IR during human neurogenesis we previously reported (Luisier et al. 2018). The cytoplasmic increase in PIR early during differentiation is likely explained by a change in the subcellular localization and/or cytoplasmic stability of (some) IRTs rather than a modulation of the splicing given the coincident stable level of PIR in the nucleus. Genes related to RNA processing and splicing are among the most affected by dynamic IR (Wong et al. 2013; Dvinge and Bradley 2015; Edwards et al. 2016; Pimentel et al. 2016; Middleton et al. 2017; Ullrich and Guigó 2020), and we previously showed that changes in IR during early MN development specifically affect RNA processing–related biological pathways (Luisier et al. 2018). Here we find that cytoplasmic (but not nuclear) IR primarily affects essential genes concerned with mRNA metabolism, whereas genes targeted by alternative exons (AltEx) are enriched in similar biological pathways in the nucleus and the cytoplasm as shown by Gene Ontology (GO) function analysis (Fig. 1D). These findings indicate that the previously reported wave of IR during MN differentiation, using whole-cell RNA sequencing, likely reflected signals from cytoplasmic IRTs.

Alongside IRTs that show temporally dynamic expression profiles, we found that as many as 3633 IRTs are detected in both the nucleus and the cytoplasm during development (CIRTs hereafter; PIR_NUCLEUS_ > 20% and PIR_CYTOPLASM_ > 15%) (Supplemental Table S2), whereas 4490 IRTs are only detected in the nucleus (NIRTs hereafter; PIR_NUCLEUS_ > 20% and PIR_CYTOPLASM_ < 5%) (Supplemental Table S3). Of the 17,729 genes that are expressed during MN development, 13% express at least one CIRT, indicating that cytoplasmic IRTs are more abundant than previously recognized.

Prior studies reported specific features associated with retained introns including higher GC content, lower intron length, and enrichment in RBP binding motifs compared with nonretained introns (Galante et al. 2004; Sakabe and de Souza 2007; Braunschweig et al. 2014; Ullrich and Guigó 2020). In line with these studies, we find that the PIR negatively correlates with the intron length and positively correlates with the GC content and the enrichment in cross-link events for 131 RBPs for which CLIP data were available (Sloan et al. 2016; Attig et al. 2018; Van Nostrand et al. 2020), both in nuclear and cytoplasmic compartments (Fig. 1E,G,I; Supplemental Fig. S1G). Additionally, we find that (1) retained introns are detected in genes containing fewer introns, and (2) the PIR positively correlates with the intronic sequence conservation score (Supplemental Fig. S1C,E,G). However, when specifically comparing the CIRTs with NIRTs, we find that the transcripts with retained introns that localize to the cytoplasm are on average longer, have lower GC content compared with their nuclear counterparts, have higher RBPs enrichment scores, and are more evolutionarily conserved (Fig. 1F,H,J; Supplemental Fig. S1D,F). Altogether, these results show that nuclear and cytoplasmic transcripts with retained introns show distinct features, including their dynamics during human motor neurogenesis, their associated biological pathways, and their molecular characteristics. These results argue against the hypothesis that cytoplasmic IRTs are intron-containing pre-mRNAs that simply “leak” from the nucleus (Yoshimoto et al. 2017).

### A spatiotemporal taxonomy reveals cytoplasmic transcripts with retained introns with distinct RBP binding profiles

**Figure 2.**
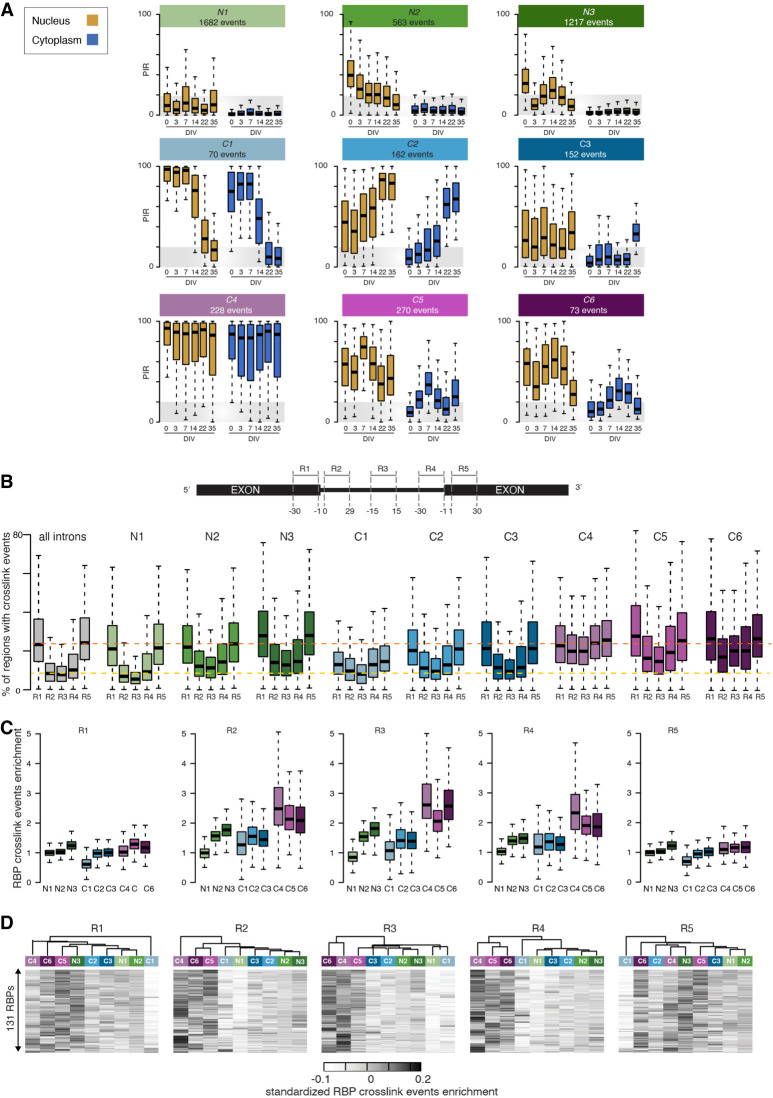
A spatiotemporal taxonomy reveals cytoplasmic IRTs with distinct RBP binding profiles. (*A*) Comparison of the nuclear and cytoplasmic PIR distributions for nine groups of retained introns showing distinct spatiotemporal dynamics during MN differentiation as identified using SVD (see Methods). N1, N2, and N3 contain introns primarily retained in the nuclear compartment, whereas the remaining six groups (C1–C6) contain introns with significant detection in the cytoplasm. Gold boxes indicate nucleus; blue boxes, cytoplasm. Gray area indicates the range of PIR values for which an intron is considered nonretained. (*B*, *top)* Schematic depicting the selected splicing regulatory regions juxtaposing the splice sites, namely, the last 30 nucleotides (nt) of the upstream exon (R1), the first 30 nt of 5′ intron region (R2), the 30 nt in the middle of the intron (R3), the last 30 nt of 3′ intron region (R4), and the first 30 nt of downstream exon (R5). (*Bottom*) Distributions of the percentage of regions in each group of introns that are mapped by at least one cross-link event for each of the available 131 RBPs. (*C*) Distribution of the enrichments in cross-link events in each of the selected regions R1, R2, R3, R4, and R5 for the available 131 RBPs across the nine categories of introns. Enrichment is obtained by dividing the fraction of regions from the group of interest with at least one cross-link event with the fraction of regions from the complete set of introns (*n* = 61,872) with a cross-link event. (*D*) Heatmaps of the enrichment scores of the cross-linking events for 131 RBPs in the R1, R2, R3, R4, and R5 regions for the nine groups of introns hierarchically clustered using Manhattan distance and Ward clustering. Data shown as box plots in which the *center* line is the median; limits are the interquartile range; and whiskers are the minimum and maximum.

Having established that nuclear and cytoplasmic IRs affect two functionally divergent mRNA subsets, we next used singular value decomposition (SVD) analysis to categorize 94,457 analyzed introns into nine groups based on their PIR spatiotemporal dynamics during MN differentiation (Fig. 2A; Supplemental Tables S4–S12). Of these, three categories are nuclear detained transcripts with retained introns (termed N1–N3 hereafter), and the other six are cytoplasmic transcripts with retained introns (termed C1–C6 hereafter), which show the following compartment-specific PIR dynamics: stable nuclear (>15%) and low cytoplasmic (<10%) PIR over time (N1), steady reduction in the nuclear PIR over time and stable low cytoplasmic PIR (N2), transient increase in nuclear PIR and stable low cytoplasmic PIR (N3), steady reduction in both nuclear and cytoplasmic PIR over time (C1), steady increase in nuclear and cytoplasmic PIR over time (C2), increase in cytoplasmic PIR only in terminal differentiation (C3), consistently high nuclear and cytoplasmic PIR over time (C4), early transient increase in nuclear and cytoplasmic PIR (C5), and late transient increase in nuclear and cytoplasmic PIR (C6). The different groups of introns together with their characteristics are summarized in Table 1. Comparing the biological pathways each of these groups is likely to impact, as revealed by GO term enrichment analysis (Supplemental Fig. S2A; Supplemental Tables S20–S28), suggests that most groups are functionally distinct, as exemplified by C1, C3, and C5.

**Table 1.**
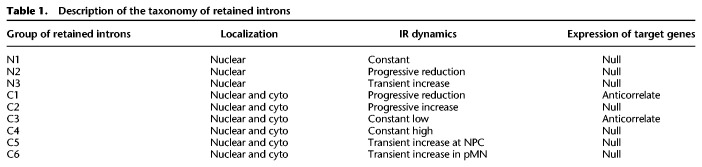
Description of the taxonomy of retained introns

Next, looking at the percentage of transcripts with retained introns per gene in each identified group revealed that IR during MN differentiation does not occur stochastically but appears to target a specific set of introns in each gene, with ∼20% of genes showing more than one retained intron in their transcripts (Supplemental Fig. S2B). This indicates that at least one additional layer of specific regulation must apply to these nine distinct IR programs as previously suggested (Ullrich and Guigó 2020). Previous studies suggest that *cis*-regulatory elements bound by *trans*-acting factors such as RBPs are likely to play a crucial role in regulating IR (Dirksen et al. 1995; Lejeune et al. 2001; Sakabe and de Souza 2007). Thus, we next sought to test whether the nine spatiotemporally distinct classes of IR we identified are associated with different combinations of *trans*-acting factors. We achieved this by using publicly available CLIP data to evaluate the cross-link events for 131 RBPs mapping to five regions we defined in relation to the acceptor and donor splice sites, namely, the last 30 nucleotides (nt) of exonic sequence upstream of the 5′ splice site (R1); the first 30 nt of intronic sequence downstream from the 5′ splice site (R2); the 30 nt in the middle of the intron, as a surrogate marker for the state of RBP binding across the deeper intron (R3); the last 30 nt of intronic sequence upstream of the 3′ splice site (R4); and the first 30 nt of exonic sequence downstream from the 3′ splice site (R5) (Fig. 2B, top; Supplemental Tables S13–S17). First, looking at the fractions of regions that are mapped by at least one cross-link event for each RBP, we find that the R1 and R5 exonic regions show the highest frequency in cross-link events across the 131 RBPs irrespective of the IR grouping, with the exception of the C1 group (Fig. 2B, bottom). These results, which are in line with previous studies showing that the splicing machinery is more likely to form across the exonic regions than across the introns for long introns (>250 nt), indicate that the nine groups of introns bear similar chances of splicing complex formation with respect to their R1 and R5 exonic regulatory regions (Robberson et al. 1990; Zhu et al. 2009). This is further supported by the finding that identically optimal splicing signals are detected among the nine groups of introns, with the exception of the C4 group (Supplemental Fig. S2C), as failure in splice site recognition (Dirksen et al. 1995; Lejeune et al. 2001; Sakabe and de Souza 2007) or decreased expression levels of splicing factors (Ullrich and Guigó 2020) have also been proposed to underlie IR. Additionally the similarly low fraction of U12 introns and high prevalence of U2 introns among the nine groups of retained introns argue in favor of similar splicing efficiency (Supplemental Fig. S2D).

Although RBPs show similarly high frequency of binding to the R1 and R5 exonic regulatory regions across the majority of the nine groups of introns, we indeed observed that the R2, R3, and R4 intronic regions display large variability in the percentages of cross-link events across the different spatiotemporal IR dynamics (Fig. 2B, bottom). Next, looking at the enrichment of RBPs mapping to each of these regions further revealed that the C4, C5, and C6 groups of cytoplasmic transcripts with retained introns are indeed specifically enriched in RBPs binding to the R2, R3, and R4 intronic regions as opposed to the R1 and R5 regions, which display as much RBP binding as the full set of introns (Fig. 2C). One of the most enriched RBPs in the R2, R3, and R4 intronic regions is UPF1 RNA helicase and ATPase (UPF1), an RNA helicase required for NMD in eukaryotes. UPF1 shows high binding occurrence across the five regions of interest for the {C4, C5, C6} groups as opposed to the other groups, where UPF1 is strictly enriched in R1 and R5 regions (Supplemental Fig. S2E). Different combinations of RBPs have been shown to coordinately regulate functionally coherent “networks” of exons and introns (Ule and Blencowe 2019). Thus, using unsupervised hierarchical clustering of the nine groups of introns based on their 131 RBP enrichment score profiles, we finally showed that {C4, C5, C6} form a coherent regulated group of introns with respect to the R2 and R4 intronic regions (Fig. 2D). Altogether, this analysis identifies a supergroup of transcripts with retained introns with a coherent RBP enrichment pattern in their intronic regulatory—as opposed to exonic regulatory—regions that are not related to splicing efficiency.

### A specific group of transcripts with retained introns in the cytoplasm with high RBP binding capacity is impacted by ALS-causing VCP mutations

**Figure 3.**
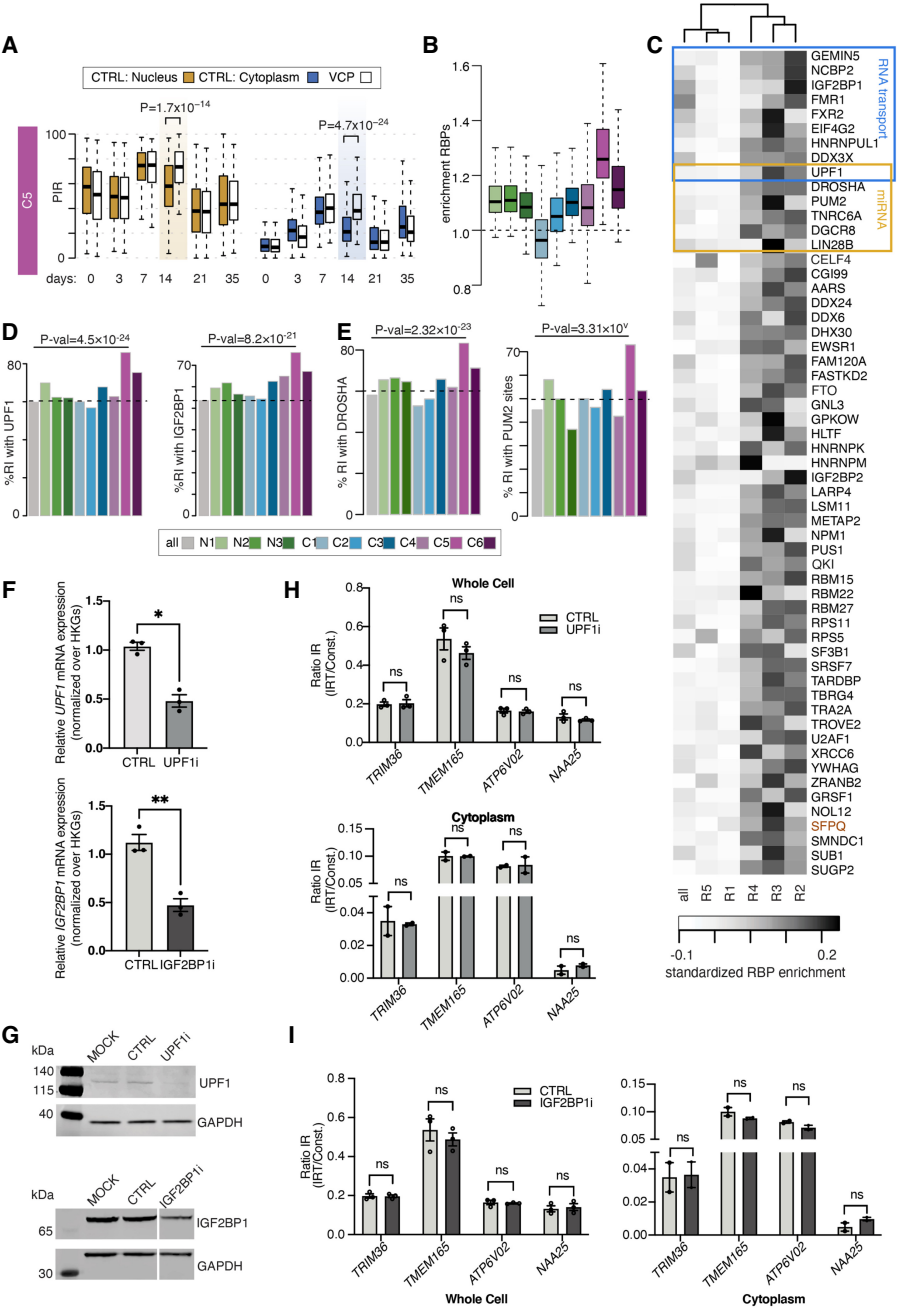
Identification of a cytoplasmic group of retained introns with a high capacity for RBP and miRNA sequestration. (*A*) Comparison of the distributions of nuclear and cytoplasmic PIR between control (colored boxes) and *VCP*^*mu*^ (white boxes) samples during MN differentiation for the C5 groups of cytoplasmic retained introns. Gold boxes indicate the time point at which the largest changes between control and *VCP*^*mu*^ samples are observed. *P*-values obtained by two-sided Welch *t*-test. (*B*) Analysis of the enrichment in binding sites for 131 RBPs across the entire retained intron for all nine categories. Enrichment is obtained by dividing the fraction of retained introns in the category of interest with a CLIP binding with the fraction of retained introns in the complete set of introns (*n* = 61,872) with a cross-linking event. (*C*) Heatmap of the enrichment score of RBP cross-linking events in the entire intronic sequence (all) and, separately, in each of the five regulatory regions of the 270 retained introns of the C5 category. Fifty-seven RBPs show >19% higher fraction of binding to the C5 group compared with the full set of introns. Blue boxes indicate RBPs involved in RNA transport; gold boxes, RBPs involved in miRNA regulation. (*D*,*E*). Percentage of retained introns with cross-link events for two RBPs involved in RNA transport (UPF1 and IGF2BP1) and two RBPs involved in miRNA regulatory pathway (DROSHA and PUM2). Dotted lines indicate mean % of retained introns with cross-linking event. *P*-value from Fisher's exact test. (*F*) Bar graphs showing qPCR analysis of *UPF1* (*top*) and *IGF2BP1* (*bottom*) relative mRNA expression levels in MNs (DIV = 21) after being transfected with siRNA targeting *UPF1* (UPF1i), *IGF2BP1* (IGF2BP1i), or a scrambled control (CTRL). Values are normalized over the geometric mean of housekeeping genes (*GAPDH*, *POLR2B*, *UBE2D3*) and expressed as fold change over mock transfected cells. Data are presented as mean ± SEM. Data are derived from three individual cell lines, one experimental block. Paired *t*-test: (*) *P* < 0.05. (*G*) SDS-PAGE/western blotting showing expression levels of UPF1 (*top*), IGF2BP1 (*bottom*), and GAPDH (*both*) in MNs (DIV = 21) transfected with siRNA targeting *UPF1*, *IGF2BP1*, a scrambled control, or mock transfected cells (MOCK). GAPDH is assayed as a loading control. (*H*) Bar graphs showing qPCR analysis of candidate C5 IR levels in whole cell (*top*) and cytoplasmic (*bottom*) MN (DIV = 21) samples upon *UPF1* knockdown. IR ratio values are calculated as levels of IRT over total transcript expression for each candidate. Data are presented as mean ± SEM. Data are derived from three individual cell lines (whole cell) and two individual cell lines (cytoplasmic), one experimental block. Multiple paired *t*-tests with significance placed at <0.05: (ns) not significant. (*I*) Same as *H*, with siRNA targeting *IGF2BP1* and whole cell (*left*) and cytoplasmic (*right*).

We previously showed widespread aberrant cytoplasmic IRTs in ALS-related *VCP* mutation (*VCP*^*mu*^)-carrying samples during MN differentiation (Luisier et al. 2018; Tyzack et al. 2021). Here, we sought to test whether *VCP*^*mu*^ affects any of the cytoplasmic groups of introns we identified in particular. Examining the dynamics of the two first principal components of cytoplasmic IR during MN differentiation in control versus *VCP*^*mu*^ samples, as captured by right singular vectors of the SVD analysis performed on the cytoplasmic PIR values, confirmed prior findings that *VCP*^*mu*^ leads to exceptionally large IR perturbations at DIV = 14 (Supplemental Fig. S3A; Tyzack et al. 2021). Further comparing the PIR distributions between control and *VCP*^*mu*^ samples in each of the six groups of identified cytoplasmic retained introns revealed that *VCP*^*mu*^ specifically impact three classes of events, namely, C5 and, to a much lesser extent, C1 and C3, whereas the other groups remain unchanged (Fig. 3A; Supplemental Fig. S3B). First, *VCP*^*mu*^-driven changes in cytoplasmic IR are unidirectional; that is, we only detect increases in IR in *VCP*^*mu*^ samples compared with control samples irrespective of PIR dynamics. Second, *VCP* mutations specifically affect groups of introns in which the PIR shows a large decrease from DIV = 7 to DIV = 14. This is in contrast to those groups of introns in which the PIR *increases* from DIV = 7 to DIV = 14, such as C2 and C6, where we find similar increase in control and *VCP*^*mu*^ samples (Supplemental Fig. S3B). Our results suggest that *VCP* mutations transiently enhance the cytoplasmic stability of IRTs before terminal differentiation, rather than affecting nuclear export. If the latter were the case, we would expect to see an equal impact on C1, C2, C5, and C6.

We previously showed that the pool of intronic sequences that accumulates in the cytoplasm of ALS neuronal cells shows high predicted binding occupancy for ALS-related mislocalized RBPs (Tyzack et al. 2021). We next characterized the entire intronic region of the nine groups of introns as opposed to the five regions defined as key regulatory regions in terms of RBP cross-link events (Supplemental Table S35). We found statistically significant differences in RBP enrichment among the nine groups (one-way ANOVA *F*-value = 145.05 and *P* < 2.2 × 10^−16^ with pairwise comparison and Tukey post hoc) with the C1 and C5 groups showing the largest and most significant differences compared with the other remaining groups (Supplemental Table S29). In line with our prior results, we found that the group of introns most affected by *VCP*^*mu*^, the C5 group, also shows the highest fraction of introns compared with all other groups with at least one cross-link event across the 131 studied RBPs compared with the full set of introns (Fig. 3B). Intron length of the C5 group is overall lower than {C1, C2, C3}, and thus, this result is unlikely to be explained by a bias in size. Of the 57 RBPs that are significantly enriched in the C5 group compared with the full set of introns (Fisher's exact test *P*-value < 0.01) (Fig. 3C–E), at least nine are key regulators of mRNA transport such as UPF1 (Graber et al. 2017; Das et al. 2019; Dalla Costa et al. 2020) and insulin like growth factor 2 mRNA binding protein 1 (IGF2BP1) (Graber et al. 2017; Das et al. 2019; Dalla Costa et al. 2020), and six RBPs are involved in miRNA regulation such as drosha ribonuclease III (DROSHA) and pumilio RNA binding family member 2 (PUM2) (Han et al. 2004; Treiber et al. 2017; Hu et al. 2018), with UPF1 being involved in both processes (Fig. 3C; Jin et al. 2009; Elbarbary et al. 2017; Park et al. 2019). These also include the splicing factor proline and glutamine rich (SFPQ) protein, which we previously showed to directly bind to its own IRT within the cytoplasm of our hiPSC ALS model, further validating our analysis in terms of the overlap between the C5 group of introns identified in this study and our previous findings of ALS-related aberrant IRTs in neurons (Tyzack et al. 2021).

The low correlation between the nuclear and cytoplasmic PIR for most cytoplasmic groups of retained introns indicates that the cytoplasmic localization and/or stabilization of these transcripts occurs through a regulated mechanism rather than at random (Supplemental Fig. S3C). Based on the interesting and unique characteristics of the C5 group of introns, we opted to solely focus on understanding the regulatory mechanisms governing this group. To test the hypothesis that RBPs with the highest enrichment in the C5 retained introns are regulating their cytoplasmic abundance, we next used siRNAs in MNs (DIV = 21) to knock down *UPF1* and *IGF2BP1*, both encoding potent regulators of mRNA transport. Following the validation of efficient knockdowns (Fig. 3F,G), we assessed levels of IR in validated C5 candidates (Supplemental Fig. S3D). In both the whole-cell (Fig. 3H,I, left) and, specifically, the cytoplasmic fraction (Fig. 3H,I, right), there was no alteration in IR for C5 candidates, suggesting that UPF1 and IGF2BP1 are not directly regulating their cytoplasmic abundance. Bioinformatic analysis revealed that ∼80% of the C5 IRTs contain at least one PTC (Supplemental Fig. S3E), making them potential targets of the NMD pathway (Kim and Maquat 2019). Following knockdown of *UPF1*, encoding a core NMD component, we observe a significant increase in growth arrest and DNA damage inducible alpha (*GADD45a*) mRNA expression levels (Supplemental Fig. S3F), a recognized target of NMD (Kurosaki et al. 2018). However, we do not observe a corresponding cytoplasmic accumulation of candidate C5 IRTs (Fig. 3H), strongly arguing against these being targets of the NMD pathway. Altogether, these results indicate that ALS-related *VCP*^*mu*^ specifically alters the cytoplasmic abundance of the C5 group of retained introns in a manner independent of either the NMD pathway or the RBP regulators of mRNA transport, for which this group of retained introns shows a high binding capacity. However, this interaction might regulate the cellular localization of these RBPs instead, as previously proposed for FUS and SFPQ (Luisier et al. 2018; Tyzack et al. 2021).

### miRNA seeds are enriched in the C5 group of intronic sequences

**Figure 4.**
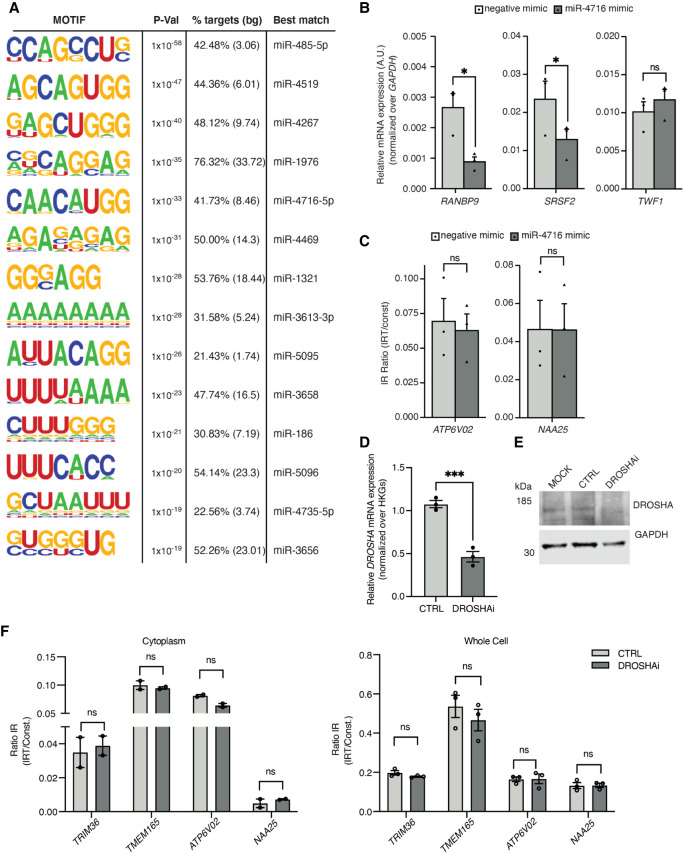
Functional investigation of the miRNA seed enrichments in the C5 group of intronic sequences. (*A*) Fourteen motifs enriched in the 270 retained introns of the C5 category identified by HOMER (Heinz et al. 2010). (*B*) Relative mRNA expression of *RANBP9* and *SRSF2* (predicted targets of miR-4716-5p) and *TWF1* mRNA in HeLa cells transfected with mimics for miR-4716-5p (miR-4716 mimic) or a scrambled negative control (negative mimic). Values are normalized over *GAPDH*. Data are presented as mean ± SEM. *t*-test: (*) *P* < 0.05. Data points represent independent experimental repeats (*n* = 3). (*C*) Analysis of IR of C5 candidates *ATP6V02* and *NAA25* in HeLa cells transfected with mimics for miR-4716-5p or a scrambled negative control. Values are calculated as levels of IRT over total transcript expression for each candidate. Data are presented as mean ± SEM. *t*-test: (*) *P* < 0.05. Data points represent independent experimental repeats (*n* = 3). (*D*) Bar graphs showing qPCR analysis of *DROSHA* relative mRNA expression levels in MNs (DIV = 21) after being transfected with siRNA targeting *DROSHA* (DROSHAi) or a scrambled control (CTRL). Values are normalized over the geometric mean of housekeeping genes (*GAPDH*, *POLR2B*, *UBE2D3*) and expressed as fold change over mock transfected cells. Data are presented as mean ± SEM. Data are derived from three individual cell lines, one experimental block. Paired *t*-test: (***) *P* < 0.0005. (*E*) SDS-PAGE/western blotting showing expression levels of DROSHA and GAPDH in MNs (DIV = 21) transfected with siRNA targeting *DROSHA*, a scrambled control, or mock transfected cells (MOCK). GAPDH is assayed as a loading control. (*F*) Bar graphs showing qPCR analysis of candidate C5 IR levels in cytoplasmic (*left*) and whole-cell (*right*) MN (DIV = 21) samples upon *DROSHA* knockdown. IR ratio values are calculated as levels of IRT over total transcript expression for each candidate. Data are presented as mean ± SEM. Data are derived from three individual cell lines (whole cell) and two individual cell lines (cytoplasmic), one experimental block. Multiple paired *t*-tests with significance placed at <0.05. (ns) not significant.

Noting that regulators of miRNA metabolism such as DROSHA, PUM2, and UPF1 are predicted to extensively bind to the C5 intronic regions, this prompted us to examine miRNA motif enrichment within these introns using HOMER (Heinz et al. 2010). This revealed significant enrichment for 14 miRNA motifs (Fig. 4A). Having found that RBPs predicted to strongly bind the C5 group of introns are likely not regulating their abundance and given the high density of miRNA seed sequences in this group, we next hypothesized that the miRNA machinery could be directly regulating their expression. miRNA-4716-5p seed sequences are among the most significantly enriched within C5 introns, and miRNA-4716-5p is expressed in human NPCs (Supplemental Fig. S5D). We first used a miRNA mimic to increase levels of miR-4716-5p in HeLa cells, where up-regulation of miR-4716-5p led to a significant reduction in transcript levels of miR-4716-5p-predicted targets serine and arginine rich splicing factor 2 (*SRSF2*) and RAN binding protein 9 (*RANBP9*) but not in twinfilin actin binding protein 1 (*TWF1*), which is not a predicted target (Fig. 4B). However, we observed that up-regulation of mir-4716-5p does not lead to an increased degradation of our candidate C5 IRTs (Fig. 4C). Similarly, we observed no change in pMNs (DIV = 18) upon treatment with miR-4716-5p mimic (Supplemental Fig. S4). Noting the possibility that the regulation of the C5 group of introns could operate through the orchestrated action of a group of miRNAs rather than a single miRNA, we next sought to suppress the overall miRNA pathway by knocking down *DROSHA* (Fig. 4D,E), a central regulator of miRNA biogenesis (Link et al. 2016), which is also enriched in the C5 intronic sequences (Fig. 3C). Loss of DROSHA, however, did not lead to significant changes in the cytoplasmic abundance of candidate C5 IRTs (Fig. 4F), indicating that attenuating the general process of miRNA interference has no significant effect on the expression levels of IRTs and raising the hypothesis that C5 IRTs may be regulating miRNA activity instead.

### Cytoplasmic IRTs of the C5 group are anticorrelated with predicted miRNA activity in health and ALS

Several lines of evidence indicate that the C5 intronic sequences do not play the canonical role of negatively regulating their transcript abundance. First, the C5 group of introns is not associated with a decrease in mRNA expression levels during MN differentiation, as opposed to the C1 group of introns, where PIR over time negatively correlates with the expression level of the genes containing the retained introns (Supplemental Fig. S5A). We indeed orthogonally validated the IRTs of both the C1 and C5 groups by RT-PCR and confirmed that although C1 IRTs correlate with reduction in mRNA expression level, C5 IRTs are not associated with such a change in their cognate spliced mRNA levels (Supplemental Fig. S5B,C). Second, *UPF1* knockdown leading to down-regulation of NMD does not result in the expected cytoplasmic accumulation of these IRTs (Fig. 3H). Thus C5 intronic sequences may, along with their abundant miRNA seeds, serve other functions beyond regulating their own gene expression level during neuronal development.

**Figure 5.**
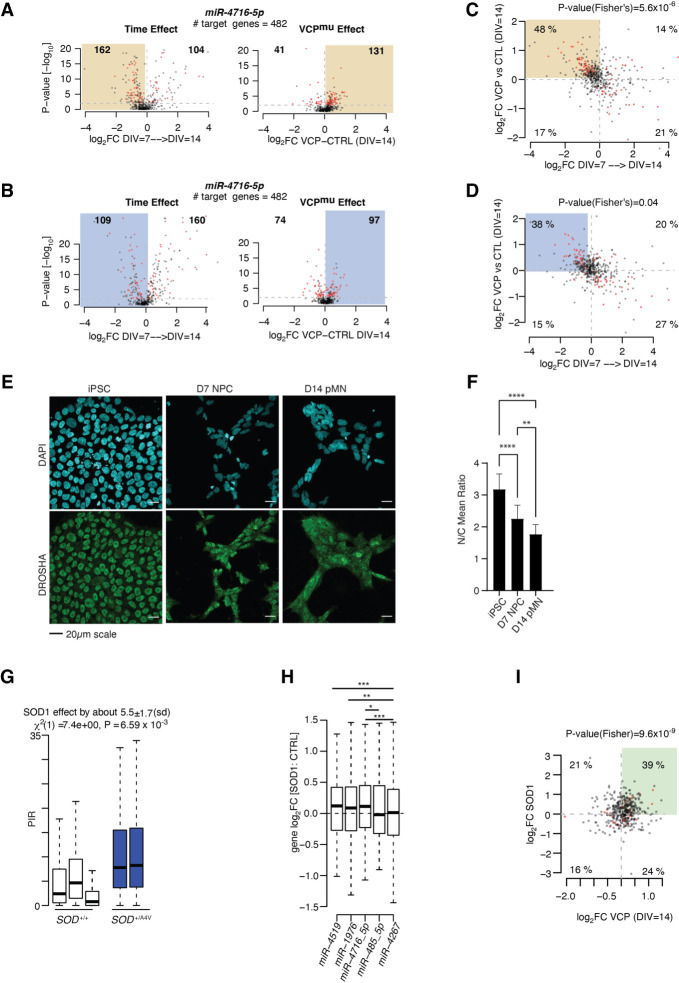
ALS-related transient accumulation in cytoplasmic retained introns correlates with reduced miRNA activity. (*A*) Volcano plots representing the effect size (i.e., log_2_(fold-change) on the *x*-axis) against the statistical significance (i.e., −log_10_(*P*-value) on the *y*-axis) of the changes in the nucleus in gene expression between two consecutive time points from DIV = 7 to DIV = 14 for control cell lines (*left*) and between control and *VCP* mutant cell lines at DIV = 14 time point (*right*) for the TargetScan predicted target genes of miR-4716-5p. (*B*) Same as *A* but for cytoplasmic gene expression changes. (*C*) Scatter plots showing the relationships of the expression changes between DIV = 7 and DIV = 14 in nuclear control samples (*x*-axis; log_2_FC) and between control and *VCP*^*mu*^ cell lines at DIV = 14 (*y*-axis; log_2_FC) of the predicted target genes for miR-4716-5p. Red color indicates genes with *P*-value < 0.01 in both comparisons. *P*-value obtained from one-side Fisher's exact test comparing the number of genes showing log_2_FC(DIV = 7 → DIV = 14) < 0 and log_2_FC(VCP vs. CTRL at DIV = 14) in the pool of miRNA predicted targets with those from the total set of genes. (*D*) Same as *C* but for cytoplasm. (*E*) Representative images of DROSHA (green) and DAPI (blue) in iPSC, NPC (DIV = 7), and pMN (DIV = 14) time points of control cells. (*F*) Nuclear-to-cytoplasmic ratio of mean DROSHA intensity is plotted for iPSC, NPC (DIV = 7), and pMN (DIV = 14) cell stages. Two-way ANOVA: (ns) nonsignificant, (*) *P* < 0.05, (**) *P* < 0.01, (***) *P* < 0.001, (****) *P* < 0.0001. Data are derived from *n* = 5 cell lines, one experimental repeat. (*G*) Boxplots displaying the distribution of PIR for the 270 introns of the C5 category in control MN (white box) and *SOD1*^*mu*^ MN samples (blue bar; *left*) (Kiskinis et al. 2014). *SOD1*^*mu*^ samples show a systematically higher proportion of IR compared with controls. Linear mixed effects analysis of the relationship between the PIR for the 270 introns and SOD1 mutation was used to account for idiosyncratic variation owing to patient differences. (*H*) Distributions of the changes in expression between *SOD1*^*mu*^ and control samples for the predicted target genes of miR-4519, miR-1976, miR-4716-5p, miR-485-5p, and miR-4267. (*I*) Scatter plot showing the relationships of the expression changes between DIV = 14 *VCP*^*mu*^ (*x*-axis; log_2_FC) and *SOD1^mu^* (*y*-axis; log_2_FC) samples of the predicted target genes for miR-4716-5p. Red color indicates genes with *P*-value < 0.01 in both comparisons. *P*-value obtained from one-side Fisher's exact test comparing the number of genes showing log_2_FC(SOD1 vs. CTRL) > 0 and log_2_FC(VCP vs. CTRL at DIV = 14) > 0 in the pool of miRNA predicted targets with those from the total set of genes. (*G*,*H*) Data shown as box plots in which the *center* line is the median; limits are the interquartile range; and whiskers are the minimum and maximum.

Previous studies proposed that stable nuclear intronic sequences act as molecular sinks to sequester and thereby functionally impair *trans*-acting factors. To investigate whether the C5 group might similarly sequester miRNAs, we next examined the predicted target gene expression profiles of the top five miRNA motifs that are significantly enriched in C5 intronic sequences (miR-4519, miR-1976, miR-4716-5p, miR-485-5p, and miR-4267) (Supplemental Tables S30–S34), specifically testing for an association between these and C5 PIR levels either in control over time from DIV = 7 to DIV = 14 or at DIV = 14 between *VCP*^*mu*^ and control samples, where C5 PIR changes are the most pronounced and significant. We find that the changes in C5 PIR level over time (decrease) and between *VCP*^*mu*^ and control samples (increase) correlate with concordant changes in the predicted target gene expression levels of miR-4716-5p, miR-4519, and, to a lower extent, miR-1976, but not miR-485-5p and miR-4267 (Supplemental Fig. S6). Gene expression changes, including statistically significant events, were clustered toward both down-regulation in control cell lines from DIV = 7 to DIV = 14 and up-regulation in *VCP*^*mu*^ cell lines compared with control cell lines at DIV = 14, with the strongest effects observed for the predicted target genes of miR-4716-5p (Fig. 5A,B; Supplemental Figs. S7, S8). These results indicate that the decrease in IR from DIV = 7 to DIV = 14 and the increase in IR in *VCP*^*mu*^ samples at DIV = 14 inversely correlate with changes in miRNA activity, respectively, in particular in the nucleus. These findings are not explained by significant differences in the expression levels of these miRNAs (Supplemental Fig. S5D). Further comparing the predicted target genes affected in both conditions revealed a significant overlap between the affected genes (*P*-value < 0.01; Fisher's exact test), thus supporting the hypothesis that the latent factor responsible for the observed changes in gene expression over time, here the miRNA-mediated gene expression regulation, is indeed perturbed in *VCP*^*mu*^ samples at DIV = 14 (Fig. 5C,D). Also, the predicted activities of miR-485-5p and miR-4267, whose target gene expression profiles did not correlate with IR level in control samples over time, also do not correlate with an increase in C5 IR in *VCP*^*mu*^ samples, thus supporting the hypothesis that the activities of specific miRNAs correlate to IR in both *VCP*^*mu*^ and control samples over time (Supplemental Fig. S6B).

We previously showed that the mislocalized SFPQ protein physically interacts with the *SFPQ* IRT, thereby supporting a model in which intronic sequences form complexes with RBPs that together localize to the cytoplasm. The finding that DROSHA is among the most enriched RBPs in the C5 intronic regions (Fig. 3C) prompted us to study the subcellular localization of DROSHA in early MN differentiation to test whether the predicted reduction in miRNA activity might relate to a reduction in DROSHA nuclear localization. In control cells, we observed a progressive decrease in the nuclear-to-cytoplasmic abundance ratio of DROSHA from DIV = 0 to DIV = 14 (Fig. 5E,F), which does not coincide with the transient increase in IR (that peaks at DIV = 7) that is characteristic of the C5 group of introns. In addition, no difference was observed between control and *VCP*^*mu*^ samples at DIV = 14 when these samples show the largest differences in PIR (Supplemental Fig. S5E,F). These findings argue against the hypothesis that the reduction in miRNA activity at the early stage of MN differentiation is induced by a reduction in nuclear abundance of DROSHA driven by the interaction with the C5 IRT sequences. Moreover, this indicates that the observed differences in miRNA target gene expression in *VCP*^*mu*^ (Fig. 5A–D) are not owing to overall perturbations in the miRNA processing machinery. Examining the predicted activities of housekeeping miRNAs indeed further indicates that changes in miRNA activity during motor neurogenesis and between control and *VCP*^*mu*^ samples target specific miRNAs (Supplemental Fig. S6E).

**Figure 6.**
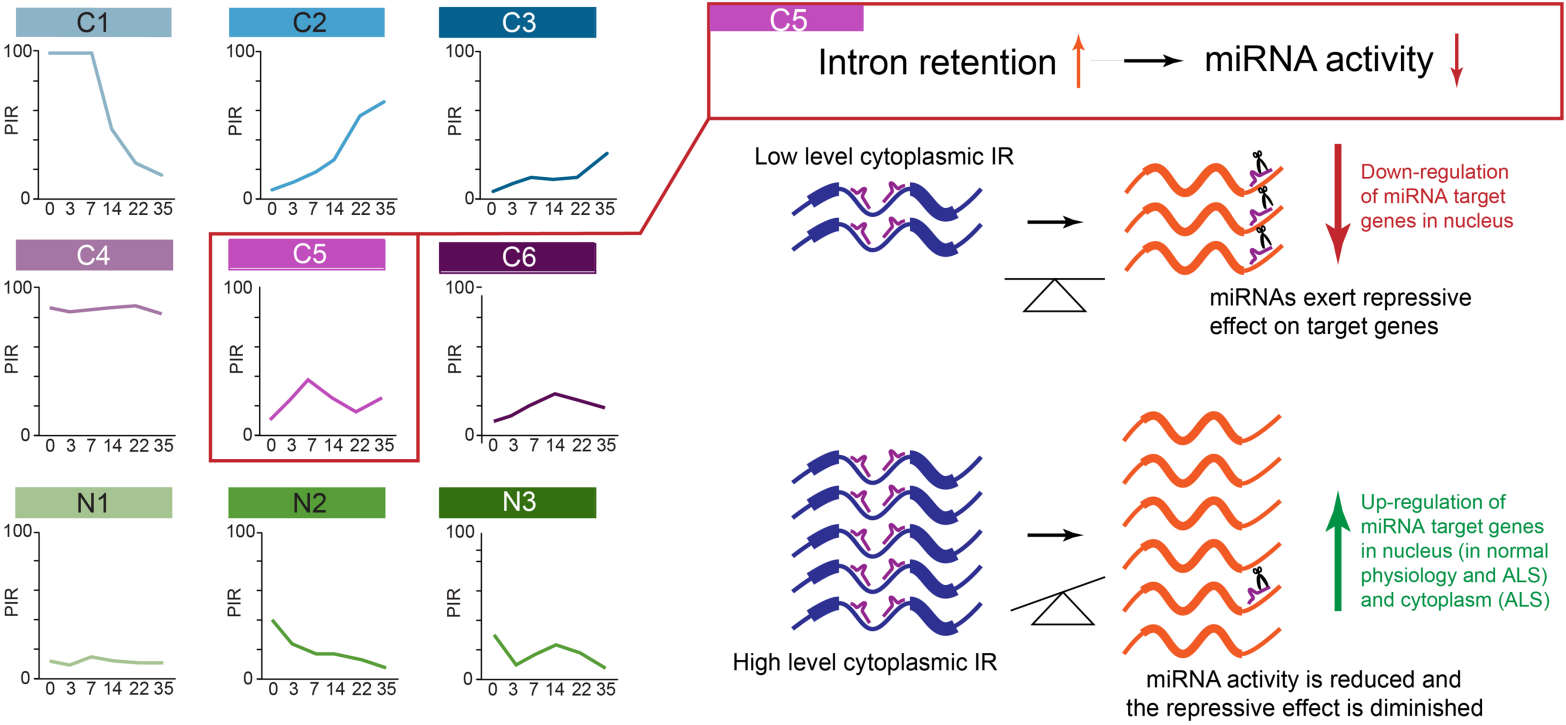
Schema of the miRNA sequestration hypothesis.

Noting that we have studied a relatively rare form of familial ALS (fALS) caused by gene mutations in *VCP* (selected as it shows the pathological hallmark of TDP-43^8^ nuclear-to-cytoplasmic mislocalization), we next sought to understand the generalizability of the association between increased IR in ALS samples and decreased predicted miRNA activity. To this end, we chose to study one of the most common forms of fALS (superoxide dismutase 1 [*SOD1*] mutations), which in contrast to *VCP* mutation-related ALS does not show TDP-43 nuclear-to-cytoplasmic mislocalization. We first looked at the PIR of the C5 group in *SOD1* mutant hiPSC-derived MNs, revealing a statistically significant increase in IR in *SOD1* (Fig. 5G). Next, looking at the changes in gene expression of the miRNA predicted target genes between *SOD1* mutant MNs and their isogenic controls, we indeed observed a significant decrease in miR-4519, miR-1976, and miR-4716-5p predicted activities compared with predicted activities of miR-485-5p and miR-4267 (*P*-value < 0.01, Welch's *t*-test) (Fig. 5H). These findings further substantiate the relevance of the correlation between increased IR and decreased miRNA activity. We also show significant overlap between the predicted target genes up-regulated at the DIV = 14 time point between *VCP*^*mu*^ and control samples and the predicted target genes up-regulated between *SOD1* and isogenic cell lines for miR-4519, miR-1976, and miR-4716-5p as opposed to miR-485-5p and miR-4267 (*P*-value < 0.01; Fisher's exact test) (Fig. 5I; Supplemental Fig. S9), further suggesting that a similar layer of regulation occurs in *SOD1* and *VCP* mutant cultures, possibly through the intronic sequences. Cumulatively, these findings support a hypothesis that C5 IRTs have a previously unrecognized function in the cytoplasm by reducing miRNA activity, potentially through sequestration (Fig. 6), as previously shown for long noncoding RNA (lncRNA) (Duss et al. 2014). This may have important roles in ALS pathogenesis and, indeed, implications for new therapeutic strategies.

## Discussion

Neuronal biology relies on complex regulation of gene expression and mRNA metabolism. AS has been shown to play a key role in this process, and IR is now recognized as the dominant mode of splicing during MN development (Braunschweig et al. 2014; Luisier et al. 2018), including cytoplasmic IR, which we recently showed to affect more than 100 transcripts in *VCP*^*mu*^-related ALS (Tyzack et al. 2021). Because nuclear IR has been the main focus of the majority of previous studies, the regulation and role of cytoplasmic IRTs remain unclear. We first show that nuclear and cytoplasmic IRs target distinct classes of mRNAs associated with particular dynamics, biological pathways, and molecular characteristics. Specifically, we find that the sequences of retained introns that localize to the cytoplasm predominantly target mRNA metabolism-related biological pathways, are more evolutionarily conserved, and show a higher capacity for RBP binding compared with their nuclearly detained counterparts. This argues against the hypothesis that intron-containing pre-mRNAs simply “leak” from the nucleus to the cytoplasm (Yoshimoto et al. 2017)—the possibility of which is further excluded experimentally—by poly(A) selection during library preparation. These findings suggest that (1) cytoplasmic localization signals for these IRTs are contained in the intronic sequences, and (2) cytoplasmic IRTs likely serve a biological function that has yet to be discovered.

We next show that MN differentiation shows complex IR spatiotemporal dynamics captured by nine distinct IR programs, three that are nuclear and six that localize to the cytoplasm. Given the timing and nucleocytoplasmic compartmental specificity of these programs, they are expected to be associated with distinct regulation. IR has been previously proposed to be the consequence of globally inefficient splicing (Ninomiya et al. 2020; Ullrich and Guigó 2020), which could be linked to several mechanisms, including the occupancy of methyl-CpG binding protein 2 (MECP2) near the splice junction (Wong et al. 2017), the expression of protein arginine methyltransferase 5 (PRMT5) (Braun et al. 2017), and relatively weak splice sites (Braunschweig et al. 2014). Here we find that the nine groups of introns show similar 5′ and 3′ maximum entropy scores as well as similarly high capacity for RBP binding in their exonic regions juxtaposed to the splice sites. These regions are where the splicing machinery is more likely to form (Robberson et al. 1990; Zhu et al. 2009) as opposed to the intronic regions themselves. These findings indicate that an overall change in splicing efficiency during MN differentiation is unlikely to be the dominant regulatory factor for most of these IR programs. Furthermore, IR during MN differentiation does not occur stochastically but appears to target a specific set of introns in each gene, thus an additional layer of specific regulation must underlie the existence of these nine distinct IR programs. Indeed, similar combinations of *trans*-acting factors are detected across two regions juxtaposed to the splices sites among three groups of cytoplasmic retained introns—{C4, C5, C6}, suggesting similar regulation. In addition, these three groups of introns show extensive capacity for RBP binding within their intronic regions juxtaposed to the splice sites compared with the full set of analyzed introns, indicating a potential regulatory mechanism in mRNA metabolism through intronic sequence binding for the {C4, C5, C6} groups of introns. The full list of retained introns for each group together with the regional RBP enrichment is freely accessible as supplemental tables, providing a rich resource for researchers across the disciplines of genomics and basic neuroscience.

We previously showed aberrant cytoplasmic IR in *VCP*^*mu*^ samples (Tyzack et al. 2021). We speculate that because *VCP* mutations lead to RBP dislocation from the nucleus in MNs (Hall et al. 2017; Luisier et al. 2018; Tyzack et al. 2019; Harley et al. 2020, 2021), this will in turn lead to less availability of these mislocalized splicing factors, which might mean that splicing is less efficient and IR occurs, with specificity imposed by relative splice site strengths. The more IR there is, the more functional sequestration of bound RBPs there will be (Tyzack et al. 2021), which sets up a self-amplifying cycle. Here we show that *VCP* mutations in particular lead to increased retention of the C5 group of introns in the cytoplasm. These cytoplasmic IR events show remarkable similarities with those we have previously reported in whole-cell samples from the same cells (Luisier et al. 2018), showing the same PIR dynamics and extensive capacity for RBP binding (Tyzack et al. 2021). Another intriguing molecular characteristic of the C5 group of introns is the enrichment for several miRNA motifs across the full length of the intron. We provide experimental evidence that RBPs and miRNAs that are predicted to strongly bind to the C5 retained introns do not regulate C5 IRT levels. This does not exclude the possibility of alterations in the translational potential of these transcripts, but this was outside the scope of this study. The precise manner in which bound miRNAs and RBPs might be regulated by the C5 IRTs themselves is of clear interest and requires further dedicated study. The absence of correlation between the nuclear and cytoplasmic PIR for several groups of CIRTs, including the C5 group, indicates that the cytoplasmic localization and/or stabilization of these transcripts occurs through a regulated mechanism rather than through errors of splicing. Furthermore, the absence of correlation between the C5 PIR level and the gene expression dynamics during MN differentiation supports our working model that intronic RNA sequences exert new, uncharacterized roles beyond gene expression regulation, particularly in the cytoplasm. Although the stable cytoplasmic localization of intronic sequences in neurons has been recognized since 2013 (Khaladkar et al. 2013), their role has remained poorly understood. One of the few studies focusing on cytoplasmic IR showed an “addressing” function for intronic RNA sequences in determining their spatial localization within cellular compartments (Sharangdhar et al. 2017). We previously proposed that cytoplasmic retained introns may act as RNA regulators of the homeostatic control of RBP localization during development and disease (Tyzack et al. 2021), which may in turn lead to loss of function of such RBPs if this delicate balance is disrupted. For example, some splicing factor proteins that are predicted to extensively bind to the C5 intronic sequences may remain sequestered upon their nuclear export and cytoplasmic localization, contributing to a transient reduction in splicing efficiency. We identified a negative correlation between the C5 PIR level and the predicted activities of miR-1976, miR-4519, and miR-4716-5p, motifs of which are enriched in the C5 intronic sequences in both our control and ALS (*VCP*^*mu*^) cultures. Furthermore, alterations in miRNA activity have previously been shown to impact on NMD efficiency, primarily through down-regulation of UPF1 activity, with the potential to alter the levels of IRTs that can harbor PTCs (Bruno et al. 2011). This does not seem to be generalizable in our system owing to the different dynamics shown by the distinct IR groups across differentiation. Moreover, even though C5 IRTs show increased UPF1 binding, these transcripts do not seem to be targeted by NMD. This instead raises the possibility that these sequences may sequester UPF1 and play a role in the decreased NMD efficiency that typically occurs in neurodevelopment, providing an interesting avenue to explore in future studies. It is noteworthy that a reduction in miRNA activity is also predicted in terminally differentiated neurons (DIV = 35) when we do not see such a significantly increased cytoplasmic IR. We speculate that the well-established increase in 3′ UTR length could contribute to this regulation, but this requires further experiments to substantiate. However, these findings were further generalized to *SOD1*-related ALS hiPSC-derived mutant MNs, supporting the hypothesis of a more generalizable functional depletion of specific miRNAs as a result of cytoplasmic intronic sequence-mediated sequestration in ALS. A reduction in miR-1976 activity, motifs of which are detected in 76% of the C5 intronic regions, is expected to occur in some sporadic ALS patients owing to a mutation (rs17162257) in its enhancer (Xie et al. 2014). Furthermore, several miRNAs, and their target genes, are recognized to be involved in the occurrence and pathophysiology of neurodegenerative diseases, including ALS (Wang and Zhang 2020; Eyileten et al. 2021; Zuo et al. 2021).

In this study, we present some interesting avenues for future research. We observed profound changes in miRNA activity in the nucleus, which are only reflected in the cytoplasm with an additional perturbation to the cellular machinery caused by *VCP* gene mutations. Previous studies have shown miRNA activity involved in the regulation of gene expression within the nucleus (Leucci et al. 2013; Laitinen et al. 2022), the work presented here suggests that this may be more extensive than previously considered. There are a number of exciting possibilities for the link between cytoplasmic IR and miRNA activity: introns might sequester miRNA in the cytoplasm, which consequently impacts the nuclear function of these miRNA, or they could act as transport shuttles that physically localize miRNA between the two compartments.

In conclusion, we propose that cytoplasmic intronic sequences function as RNA regulators in the homeostatic control of RBP localization/function and miRNA activity during MN development. Furthermore, we propose that a group of intronic sequences that accumulate in the cytoplasm of *VCP* mutant cultures, as previously shown (Tyzack et al. 2021), acts as molecular sponges for miRNA, thus resulting in elevated expression of their target genes. Such a titration model will ultimately be determined by the stoichiometry between C5 IRTs and miRNAs (Jens and Rajewsky 2015). Such a mechanism would have potential implications for ALS pathogenesis and the development of therapies for this devastating and incurable disease.

## Methods

### Compliance with ethical standards

Informed consent was obtained from all patients and healthy controls in this study. Experimental protocols were all performed according to approved regulations and guidelines by UCLH's National Hospital for Neurology and Neurosurgery and UCL's Institute of Neurology joint research ethics committee (09/0272).

### RNA sequencing data

We obtained paired-end poly(A)-stranded RNA-seq libraries prepared from fractionated nucleus and cytoplasm obtained from six distinct stages of MN differentiation from control and *VCP*^*mu*^ samples (iPSC, and days 3, 7, 14, 21, and 35) (Supplemental Table S1) from a previously published study (obtained from the NCBI Gene Expression Omnibus [https://www.ncbi.nlm.nih.gov/geo/] under accession number GSE152983) (Tyzack et al. 2021). The cellular material was derived from four healthy controls and two ALS patients with *VCP* mutations: R155C and R191Q (95 samples from six time points and two genotypes [healthy and *VCP*^*mu*^-related ALS], four clones from four different healthy controls and three clones from two *VCP*^*mu*^ patients). Further details of the RNA-seq quality control, as well as samples, can be found in Supplemental Table S2 of our previous paper (Tyzack et al. 2021). We also obtained paired-end RNA sequencing reads derived from one independent study on familial form of ALS caused by mutant *SOD1* (*n* = 5; two patient-derived SOD1A4V and three isogenic control MN samples in which the mutation has been corrected; Hb9 FACS purified MNs, GEO: GSE54409) (Kiskinis et al. 2014).

### Transcript and gene expression analysis

kallisto (Bray et al. 2016) was used to (1) build a transcript index from the GENCODE hg38 release *Homo sapiens* transcriptome (-k 31), (2) pseudoalign the RNA-seq reads to the transcriptome, and (3) quantify transcript abundances (-b 100 -s 50—rf-stranded). Subsequent analysis was performed with the R statistical package version 3.3.1 (2016) and Bioconductor libraries version 3.3 (R Core Team 2022). kallisto outputs transcript abundance, and thus, we calculated the abundance of genes by summing up the estimated raw count of the constituent isoforms to obtain a single value per gene. For a given sample, the histogram of log_2_ gene count is generally bimodal, with the modes corresponding to nonexpressed and expressed genes. Reliably expressed genes/transcripts for each condition (*VCP*^*mu*^ or control at days 0, 3, 7, 14, 22, and 35 in each fraction) were next identified by fitting a two-component Gaussian mixture to the log_2_ estimated count gene/transcript data with R package mclust (Fraley et al. 2012); a pseudocount of one was added before log_2_ transformation. A gene/transcript was considered to be reliably expressed in a given condition if the probability of it belonging to the nonexpressed class was under 1% in each sample belonging to the condition; 18,834 genes and 102,047 transcripts were selected based on their detected expression in at least one of the 24 conditions (i.e., six different time points of lineage restriction for control and *VCP*^*mu*^ in nuclear and cytoplasm). Next, we quantile normalized the columns of the count matrices with R package limma (Boldstad et al. 2003). For differential gene expression analysis, we ran Sleuth (Pimentel et al. 2017).

### Splicing analysis

The identification of all classes of AS events in MN differentiation was performed with the Vertebrate Alternative Splicing and Transcription Tools (VAST-TOOLS) toolset, which works synergistically with the VastDB web server, a collection of species-specific AS library files (Irimia et al. 2014). Paired-end stranded RNA-seq reads were first aligned with VAST-TOOLS against the *H. sapiens* hg38, Hs2 assembly from VastDB with the scaffold annotation Ensembl v88. This contains 74,030 exon skipping events, 153,119 IR events, 474 microexon events, 20,812 alternative 3′ UTR events, and 15,804 alternative 5′ UTR events. We then merged files from identical samples but different lanes together and then performed differential splicing analysis over time either for the control or for the *VCP* mutant samples separately using the vast-tools diff command, which takes into account the different biological replicates. We then imported the result tables into R. For an AS event to be considered differentially regulated between two conditions, we required that the minimum average ΔPSI (between the paired replicates) be at least 15% and that the transcript targeted by the splicing event in question to be reliably expressed in all samples from the conditions compared, namely, enough read coverage in all samples of interest.

### GO enrichment analysis

GO enrichment analysis was performed using classic Fisher's exact test with topGO Bioconductor package (Alexa and Rahnenfuhrer 2022). Only GO terms containing at least 10 annotated genes were considered. A *P*-value of 0.05 was used as the level of significance. On the figures, top significant GO terms were manually selected by removing redundant GO terms and terms that contained fewer than five significant genes.

### Mapping and analysis of CLIP data

To identify RBPs that bind to retained introns, we examined iCLIP data for 21 RBPs (Attig et al. 2018) and eCLIP data from K562 and HepG2 cells for 112 RBPs available from ENCODE (Sloan et al. 2016; Van Nostrand et al. 2020). Before mapping the reads, adapter sequences were removed using cutadapt v1.9.dev1 (Martin 2011), and reads <18 nt were dropped from the analysis. Reads were mapped with STAR v2.4.0i (Dobin et al. 2013) to the UCSC hg19/GRCh37 genome assembly. The results were lifted to hg38 using liftOver (Hinrichs et al. 2006). To quantify binding to individual loci, only uniquely mapping reads were used.

### Analysis of *cis*-acting features

MaxEntScan (Yeo and Burge 2004) was used to calculate maximum entropy scores for 9-bp 5′ splice sites and 23-bp 3′ splice sites. Intron lengths and GC content were calculated using the hg38 human genome assembly. The intronic enrichment for the RBP binding site was obtained by computing the proportion of cross-link events mapping to retained intron compared with nonretained introns of the same genes, accounting for intron length. These were defined in relation to the acceptor and donor splice sites, namely, the last 30 nt of exonic sequence upstream of the 5′ splice site (R1), the first 30 nt of intronic sequence downstream from the 5′ splice site (R2), the 30 nt in the middle of the intron (R3), the last 30 nt of intronic sequence upstream of the 3′ splice site (R4), and the first 30 nt of exonic sequence downstream from the 3′ splice site (R5). These regions were defined based on the past studies of the Nova RNA splicing map (Ule et al. 2006), which has been determined by the positioning of conserved YCAY clusters and by the binding sites identified by HITS-CLIP as previously reported (Cereda et al. 2014). The nucleotide-level evolutionary phastCons scores for multiple alignments of 99 vertebrate genomes to the human genome were obtained from UCSC (Siepel et al. 2005; Pollard et al. 2010), and a median score was derived for each individual intron and defined regions of interest. The RBP cross-link event enrichment scores in each region of interest or in each group of introns were obtained by dividing the fractions of introns in a given group over the fractions in the full list of introns that show at least one cross-link event for a given RBP in the defined region or across the full intronic region. miRNA motif analysis within the introns was obtained using HOMER (Heinz et al. 2010), selecting for the significant motifs (*P*-value < 0.05). The target genes for the top five miRNAs were then obtained using TargetScan (Agarwal et al. 2015). U12- and U2-type of introns annotations were obtained from IntronDB database (Moyer et al. 2020).

### Analysis of the relationship between the PIR and characteristic features of retained introns

To test the relationship between the PIR in the nucleus or the cytoplasm and characteristic features of intronic sequences, we used the analysis of variance comparing a full linear model, which predicts the maximum PIR across all nuclear/cytoplasmic samples using five characteristics (the logit of intron length, the intron GC content, the conservation score of the intron, enrichment of the intron in RBP peaks, and the number of introns in the target gene), with a reduced linear model in which the characteristic of interest is removed.

### Spatiotemporal taxonomy of the retained introns

We performed SVD on the PIR cytoplasmic versus nuclear values of 94,457 introns in *n* = 48 cytoplasmic samples and *n* = 47 nuclear samples across the five distinct stages of MN differentiation from healthy controls and *VCP* mutants. We analyzed 94,457 introns out of the 153,119 annotated introns in VAST-TOOLS given their overlap with reliably expressed genes. We then selected one component that maximally captures variance in PIR. To visualize the right singular vectors $\{{\vec{v}}_{k}\}$, we plotted the PIR on the vertical axis as a function of the time corresponding to each sample on the horizontal axis, coloring all samples corresponding to healthy controls with filled circles and those corresponding to *VCP* mutants with empty circles. Next, we identified introns whose PIR profiles correlated (Pearson's correlation between individual intron PIR profile and right singular vectors) and contributed (projection of each individual intron PIR profile onto right singular vectors) most strongly (either positively or negatively) to the profile of the singular vectors. To identify representative introns for each singular vector, events were ranked according to both projection and correlation scores. The highest (most positive scores in both projection and correlation) and lowest (most negative scores in both correlation and projection) motifs were selected for each singular vector using *k*-means clustering.

### RNA extraction

RNA was extracted using the Maxwell RSC simplyCells RNA kit (Promega) and the Maxwell RSC instrument (Promega), according to the manufacturer's instructions. Contaminating genomic DNA was removed by DNase I digestion during the RNA extraction. RNA concentration and purity ratios were determined by NanoDrop (Thermo Fisher Scientific).

### RT-qPCR validations

A RevertAid first-strand cDNA synthesis kit (Thermo Fisher Scientific) was used to synthesize cDNA from whole-cell RNA samples or RNA samples derived from nuclear and cytoplasmic fractions of the relevant time points. Typically, 200–500 ng RNA was reverse-transcribed per each reaction that used random hexamers. Appropriate dilution of the cDNA was then used in qPCR reactions containing PowerUp SYBR Green master mix and primer pairs, using the QuantStudio 6 flex real-time PCR system (Applied Biosystems). Reactions omitting reverse transcriptase and containing no template were used as controls. Primers used are listed in Supplemental Table S18, and each primer pair was determined to have 90%–110% efficiency. Relative mRNA expression levels between control and mutant cells and/or between relevant time points were quantified using ΔΔCT method, with the geometric mean of glyceraldehyde-3-phosphate dehydrogenase (*GAPDH*), RNA polymerase II subunit B (*POLR2B*), and ubiquitin conjugating enzyme E2 D3 (*UBE2D3*) used as a reference for normalization for total-cell RNA samples and the geometric mean of nitrilase 1 (*NIT1*) and nuclear transcription factor, X-box binding 1 (*NFX1*), used as a reference for normalization for fractionated samples. Reference genes were selected as their gene expression does not change significantly between time points and/or in a compartment-specific manner. To assess the percentage of retained introns, primers were designed to target relevant retained intron and constitutive regions of the mature transcript (to assess total gene expression). The percentage of IR (IR ratio) was calculated by dividing the ΔCT value of retained intron and constitutive transcript. Data were plotted using RStudio software, version 4.0.3 (RStudio Team 2020).

### miRNA expression analysis

Total RNA including small RNAs was extracted from relevant time points and/or cell lines using a mirVana miRNA isolation kit (Invitrogen). RNA quantification and its 260/280 ratio were assessed using the NanoDrop (Thermo Fisher Scientific). Poly(A) tailing and reverse transcription of mature miRNAs were performed using a miRCURY LNA RT kit (Qiagen), with 100 ng of total RNA as input. Reverse-transcribed cDNA was quantified using miRCURY LNA SYBR Green dye, specifically designed primers (miRCURY LNA miRNA PCR assays), and the QuantStudio 6 flex real-time PCR system (Applied Biosystems). Data were quantified using the ΔΔCT method, with U6 snRNA as a reference gene for normalization, and were plotted using RStudio software, version 4.0.3 (RStudio Team 2020). For primer sequences, see Supplemental Table S18.

### Cell fractionation

Biochemical subcellular nuclear–cytoplasmic fractionation was performed as previously (Tyzack et al. 2021). Briefly, this was achieved using the cell fractionation buffer of the Ambion PARIS kit (Thermo Fisher Scientific) and its general instructions to recover the cytoplasmic fraction and a nuclear pellet, which was then lysed using an 8 M nuclear lysis buffer (50 mM Tris-HCL at pH 8, 100 mM NaCl, 0.1% SDS, and 1 mM DTT) prepared in house. Both lysis buffers were supplemented with 0.1 U/μL RiboLock RNase inhibitor (Thermo Fisher Scientific) and HALT protease inhibitor complex (Thermo Fisher Scientific). Briefly, the cells were washed in PBS, and the cell pellet was lysed in an appropriate volume of cell fractionation buffer on ice for 10 min. Nuclei were pelleted at 500*g* for 2 min, and the cytoplasmic supernatant was carefully removed into a fresh microcentrifuge tube, taking care not to disturb the pellet. The cytoplasmic supernatant was cleared of any debris and particles by centrifugation at maximum speed, and the final supernatant was used as cytoplasm. Nuclei were washed once in the cell fractionation buffer and then lysed in the 8 M nuclear lysis buffer on ice for 30 min. The nuclear fraction was finally homogenized through a QIAshredder (Qiagen) to fragment chromatin and reduce viscosity. Nuclear and cytoplasmic fractions were then subjected to RNA extraction as above.

### Protein extraction and SDS-PAGE/western blotting

Proteins were extracted using RIPA lysis and extraction buffer (Thermo Fisher Scientific) supplemented with HALT protease inhibitor complex (Thermo Fisher Scientific) by a 10-min incubation on ice, followed by 10 cycles of 30-sec on/30-sec off sonication using a Bioruptor pico sonication device (Diagenode), a subsequent 30-min incubation on ice, and a 5-min clearing step at maximum centrifugation speed. Proteins were quantified using DC protein assay (Bio-Rad), according to manufacturer's instructions. An appropriate volume of 4× NuPAGE LDS sample buffer was added to 10 μg of protein sample as well as DTT as a reducing agent to a final concentration of 50 mM. Protein samples were then denatured for 10 min at 70°C, loaded onto an Invitrogen NuPAGE 4%–12% Bis-Tris protein gel (Thermo Fisher Scientific), and run at 180 V for 1–2 h in NuPAGE MOPS running buffer (Thermo Fisher Scientific). Either PageRuler prestained protein ladder, 10–180 kDa (Thermo Fisher Scientific 26616), or PageRuler Plus prestained protein ladder, 10–250 kDa (Thermo Fisher Scientific 26619), was run alongside as the marker of protein molecular weight. Proteins were blotted onto a 0.45-µm nitrocellulose membrane using an XCell II blot module wet transfer apparatus at 330 mA for 2 h. Membranes were blocked in TBS containing 0.1% Tween-20 (TBST) and 5% milk for 1 h at room temperature. Incubation with an appropriate dilution of the primary antibody in TBST was performed overnight at 4°C. Three 10-min washes were then performed in TBST, followed by incubation with an appropriate fluorescent IRDye 680 or 800CW secondary antibody (LI-COR Biosciences) at 1:10,000 dilution in TBST for 1 h at room temperature. After a further three 10-min washes in TBST, the signal was imaged using an Odyssey CLx infrared imaging system (LI-COR Biosciences). Antibodies used in this study are detailed in Supplemental Table S19.

### miRNA overexpression

miRNA mimics (hsa-miR-4716-5p mirVana miRNA mimic, Thermo Fisher Scientific assay ID MC22455) were used to assess the effect of miRNA overexpression on IR. HeLa cells were seeded to ∼30% confluency, whereas 5 × 10^5^ of pMNs (DIV = 18) were plated per well of a 12-well format for transfection. Lipofectamine RNAiMAX was used as a transfection reagent according to manufacturer's instructions, whereby miRNA mimics were used at a 5-nM final concentration. Scramble RNA was used as a negative control. The cells were harvested for RNA extraction 24/48 h posttransfection. miRNA target genes whose response to the mimic was assessed were selected based on the predicted hsa-miR-1976 target score and their expression in the relevant cell type, using miRDB (Chen and Wang 2020).

### RBP knockdown

siRNAs targeting human *UPF1*, *DROSHA*, and *IGF2BP1* were obtained from Horizon (pool of four siRNAs, ON-TARGETplus Human UPF1 siRNA, L-011763-00-0005; pool of four siRNAs, ON-TARGETplus human DROSHA siRNA, L-016996-00-0005; pool of four siRNAs, ON-TARGETplus human IGF2BP1 siRNA, L-003977-00-0005). Patterned MNs (DIV = 18) were plated at a density of 1 × 10^6^ cells/well in six-well plates. Cells were transfected using Lipofectamine RNAiMAX (Thermo Fisher Scientific), according to the manufacturer's instructions, with 75 nmol of appropriate siRNA used per transfection. Scramble RNA was used as a negative control, whereas the mock sample was treated with the empty transfection reagent. After overnight incubation, the media were changed into N2B27 media containing 0.1 µM compound E to allow the cells to differentiate into MNs. The cells were differentiated over the course of 72 h and then harvested for either total-cell RNA and protein extraction or subcellular nucleo-cytoplasmic fractionation followed by RNA extraction, as detailed above. At the point of collection, cells are DIV = 21.

### DROSHA immunocytochemistry

DROSHA subcellular localization was assessed by DROSHA immunocytochemistry at relevant MN differentiation time points. iPSCs were plated onto coverslips in 12-well plates at a 50% confluency, whereas NPCs were plated onto coverslips the day before each time point in appropriate media containing 10 µM ROCK inhibitor. The media were changed to exclude ROCK 4 h before fixing. Cells were fixed using 4% paraformaldehyde in PBS for 15 min at room temperature and washed three times in PBS, and the cells were stored in PBS at 4°C before all time points were collected. All the following steps were performed at room temperature unless otherwise indicated. Cells were permeabilized for 10 min in PBS containing 0.3% Triton X-100, which was followed by three 5-min washes in PBS and a 30-min blocking step performed using 3% BSA in PBS. Cells were then incubated with a rabbit recombinant anti-DROSHA antibody (EPR12794; Abcam, ab183732) at 1:400 dilution in PBS 3% BSA overnight at 4°C in a humidified chamber. Cells were subjected to three 5-min washes in PBS and incubated with a donkey antirabbit IgG Alexa Fluor 488 secondary antibody (ab150073) and DAPI at 1:1000 in PBS, to stain the nuclei, for 45 min in a humidified dark chamber protected from light. Cells were finally subjected to three 5-min washes in PBS, and the coverslips were mounted onto microscope slides using Dako mounting medium (Agilent) and left to dry for 15 min at room temperature and overnight at 4°C before the images were taken using a Zeiss invert 880 confocal microscope. A maximum projection of the images was taken from the Z stack. Using Stardist, an image J plugin, the nuclei were identified from the DAPI channel to make a nuclear mask. Using CellProfiler (Carpenter et al. 2006), the nuclei were then filtered to remove small dead cells and debris. The nuclear mask was expanded by 10 pixels, and this region was then defined as the cytoplasm. The mean intensities for DROSHA for each compartment were calculated using the defined nuclear and cytoplasmic masks. Finally, the DROSHA Nuclear-to-cytoplasmic ratio was calculated by dividing the mean of all the nuclei in a field by the mean of all the cytoplasm in that field. Data quantification was performed using GraphPad Prism.

## Supplementary Material

Supplemental Material

## Footnotes

8 TDP-43 is an alias of the HUGO Gene Nomenclature Committee-approved symbol TARDBP.
