# Supplementary material for: Physiological intron retaining transcripts in the cytoplasm abound during human motor neurogenesis
Source: Genome Res. 2022 Oct;32(10):1808–25. doi: 10.1101/gr.276898.122 (PMC9712626; doi:10.1101/gr.276898.122)
Supplement: Supplemental Material [file supp_32_10_1808__DC1.html]

Physiological intron retaining transcripts in the cytoplasm abound during human motor neurogenesis — Physiological intron retaining transcripts in the cytoplasm abound during human motor neurogenesis — Supplemental Material 

# Physiological intron retaining transcripts in the cytoplasm abound during human motor neurogenesis

## Supplemental Material

- Supplemental\_Tables\_S1\_to\_S35.xlsx
- Supplemental\_Material.pdf
