# Supplementary material for: Physiological intron retaining transcripts in the cytoplasm abound during human motor neurogenesis
Source: Genome Res. 2022 Oct;32(10):1808–25. doi: 10.1101/gr.276898.122 (PMC9712626; doi:10.1101/gr.276898.122)
Supplement: Supplemental Material [file supp_gr.276898.122_Supplemental_Material.pdf]

*Supplemental Material*

# Physiological intron retaining transcripts in the cytoplasm abound during human motor neurogenesis

*Marija Petrić Howe[1,2§], Hamish Crerar[1,2§], Jacob Neeves[1,2], Jasmine Harley[1,2], Giulia E. Tyzack[1,2], Pierre Klein[1,3], Andres Ramos[1,3] Rickie Patani[1,2,#], Raphaëlle Luisier[4,#]*

*[1]The Francis Crick Institute, 1 Midland Road, London NW1 1AT, UK; [2]Department of Molecular Neuroscience, UCL Institute of Neurology, Queen Square, London, UK; [3]Research Department of Structural and Molecular Biology, University College London, Darwin Building, Gower Street, London, WC1E 6XA, UK. [4]Idiap Research Institute, Genomics and Health Informatics, Martigny, Switzerland;*

*[§]These authors contributed equally to this work.*

*[#]These authors contributed equally to this work.*

## Supplementary Figure 1

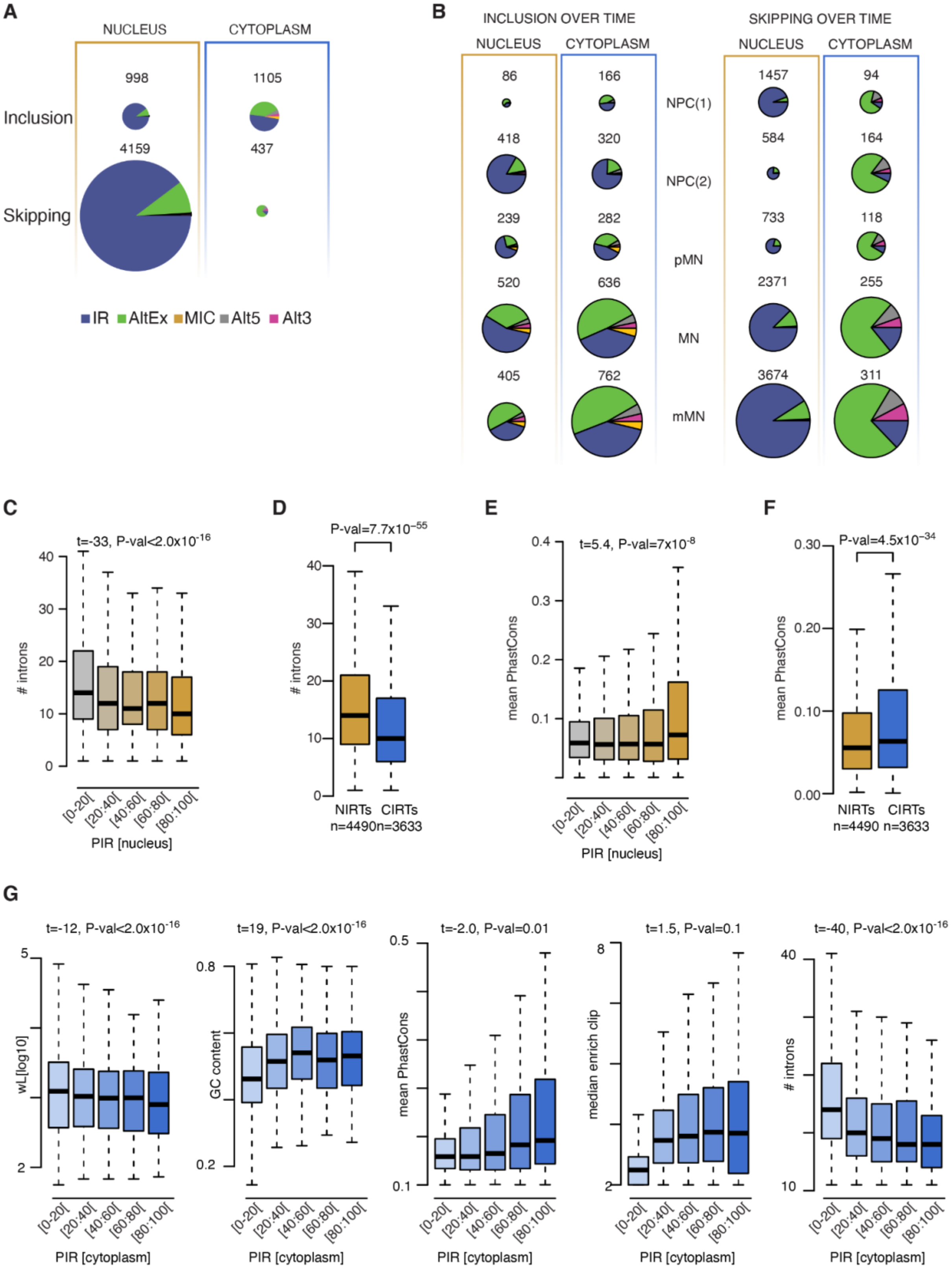


**Supplemental Figure S1 | A.** Pie charts representing proportions of included (*upper*) and skipped (*lower*) splicing events in healthy control samples pooled from

the five stages of motor neurogenesis in nuclear (*left*) and cytoplasmic (*right*) fractions. Total number of events are indicated above the charts. Intron retention (IR); alternative exon (AltEx); microexons (MIC); alternative 5′ and 3′ UTR (Alt5 and Alt3). **B.** Pie charts representing distributions of included and skipped splicing events in healthy control samples at distinct stages of motor neurogenesis compared to iPSCs or previous time-point. Induced-pluripotent stem cells (iPSC); neural precursors (NPC); ventral spinal cord precursor motor neurons (pMN); post-mitotic but electrophysiologically inactive motor neurons (MN); electrophysiologically active MNs (mMN). **C, E.** Analysis of the relationship between the percent intron retention (PIR) in the nucleus and the number of introns per gene (**C**), and the retained intron average conservation scores (**E**). Retained introns are grouped in five categories of increasing level of retention in the nucleus as indicated on the *x*-axis. P-values obtained from analysis of variance comparing the full model of the logit of maximum IR across all nuclear samples according to the five characteristics with the reduced model removing the characteristic of interest. **D, F.** Comparison of number of introns per gene (**D**) and the conservation scores (**F**) between nuclear and cytoplasmic retained introns. Nuclear retained introns are defined as those exhibiting >20% IR in nuclear fraction and <5% IR in cytoplasmic fraction. Cytoplasmic retained introns are defined as those exhibiting >20% IR in nuclear fraction and >15% IR in cytoplasmic fraction. P-values obtained from Mann-Whitney U test. **G.** Analysis of the relationship between the percent intron retention (PIR) in the cytoplasm and the intron length, the GC content in %, the number of introns per gene, the retained intron average conservation scores and the median enrichment for RBP binding site compared to the non-retained introns of the same gene. Retained introns are grouped in five categories of increasing level of retention in the cytoplasm as indicated on the *x*-axis. Data shown as box plots in which the center line is the median, limits are the interquartile range and whiskers are the minimum and maximum.

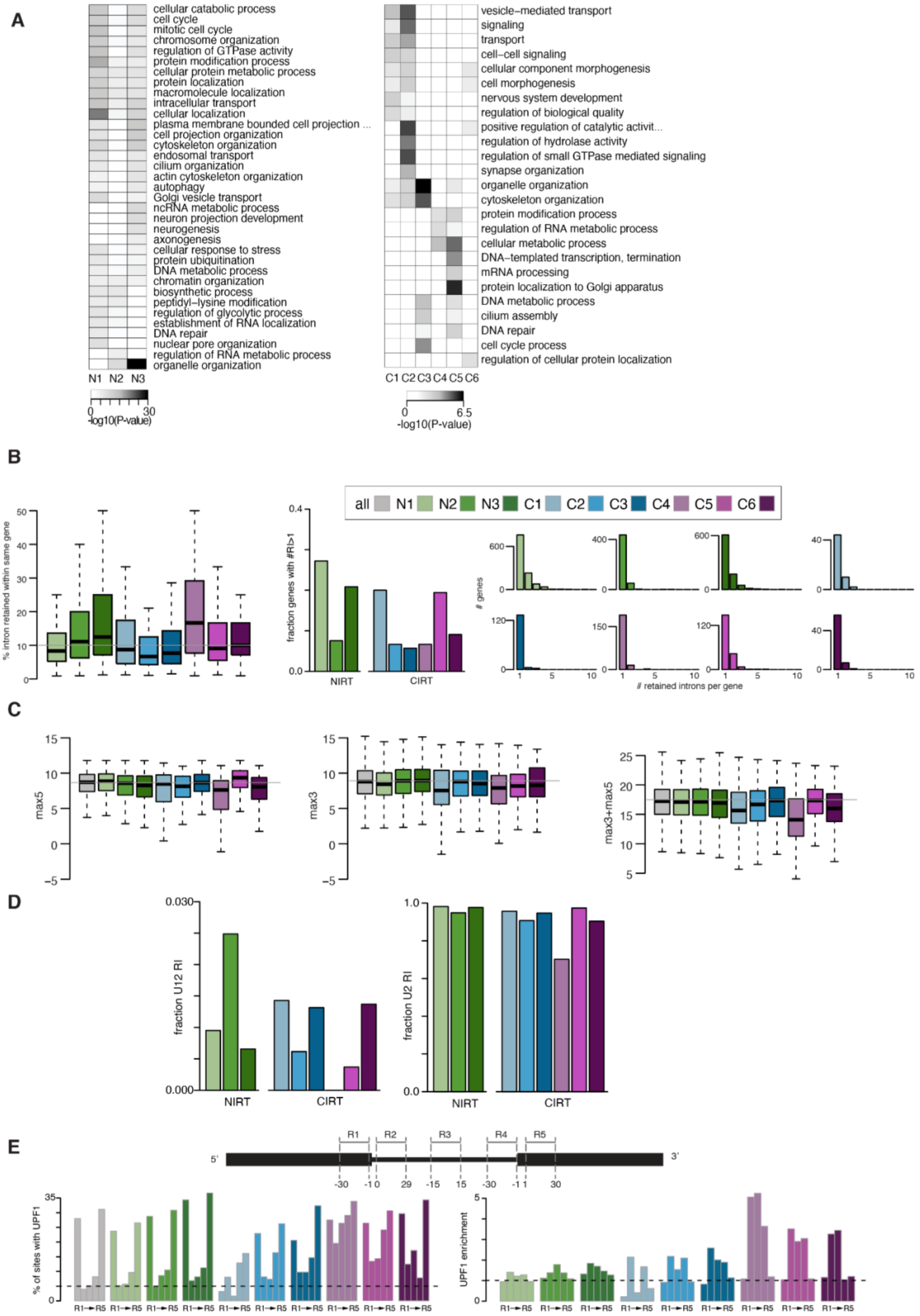
A
cellular catabolic process
cell cycle
mitotic cell cycle
chromosome organization
regulation of GTPase activity
protein modification process
cellular protein metabolic process
protein localization
macromolecule localization
intracellular transport
cellular localization
plasma membrane bounded cell projection ...
cell projection organization
cytoskeleton organization
endosomal transport
cilium organization
actin cytoskeleton organization
autophagy
Golgi vesicle transport
ncRNA metabolic process
neuron projection development
neurogenesis
axonogenesis
cellular response to stress
protein ubiquitination
DNA metabolic process
chromatin organization
biosynthetic process
peptidyl−lysine modification
regulation of glycolytic process
establishment of RNA localization
DNA repair
nuclear pore organization
regulation of RNA metabolic process
organelle organization
N1 N2 N3
0 30
-log10(P-value)
vesicle−mediated transport
signaling
transport
cell−cell signaling
cellular component morphogenesis
cell morphogenesis
nervous system development
regulation of biological quality
positive regulation of catalytic activit...
regulation of hydrolase activity
regulation of small GTPase mediated signaling
synapse organization
organelle organization
cytoskeleton organization
protein modification process
regulation of RNA metabolic process
cellular metabolic process
DNA−templated transcription, termination
mRNA processing
protein localization to Golgi apparatus
DNA metabolic process
cilium assembly
DNA repair
cell cycle pore process
regulation of cellular protein localization
C1 C2 C3 C4 C5 C6
0 6.5
-log10(P-value)
B
% intron retained within same gene
fraction genes with #RI>1
NIRT
CIRT
all N1 N2 N3 C1 C2 C3 C4 C5 C6
# genes
# retained introns per gene
C
max5
max3
max3+max5
D
fraction U12 RI
fraction U2 RI
NIRT
CIRT
E
R1 R2 R3 R4 R5
5'
3'
-30 -1 0 29 -15 15 -30 -1 1 30
% of sites with UPF1
UPF1 enrichment
R1→R5

**Supplemental Figure S2 | A.** Gene Ontology Enrichment Analysis of the genes exhibiting intron retention of a specific dynamic over time. **B.** (*left*) Percentage of retained introns per gene for the genes targeted by intron retention in each group; (*center*) fraction of genes with more than one retained intron in each group; (*right*) distribution of the number of retained introns per gene in each group. **C.** Distribution of the maximum entropy scores for 9-bp 5′ (*left*), 23-bp 3′ (*center*) and the sum of 9-bp 5′ and 23-bp 3′ splice sites (*right*) for the 9 categories of introns as obtained from MaxEntScan (1). **D.** Fractions of U12 (*left*) and U2 (*right*) retained introns for the 9 categories of introns as annotated in IntronDB (2). **E.** Percentage of introns with UPF1 regional cross-linking events (*left*) and UPF1 regional cross-linking enrichment (*right*) for each splicing regulatory region: the last 30 nts of the upstream exon (R1), the first 30 nts of 5' intron region (R2), the 30 nts in the middle of the intron (R3), the last 30 nts of 3' intron region (R4), and the first 30 nts of downstream exon (R5) for the 9 categories of introns. Dashed lines indicate the average percentage of all 61872 analysed introns with a CLIP binding (*left*) and the one-fold enrichment (*right*) in the intronic regulatory regions (R2, R3, R4).

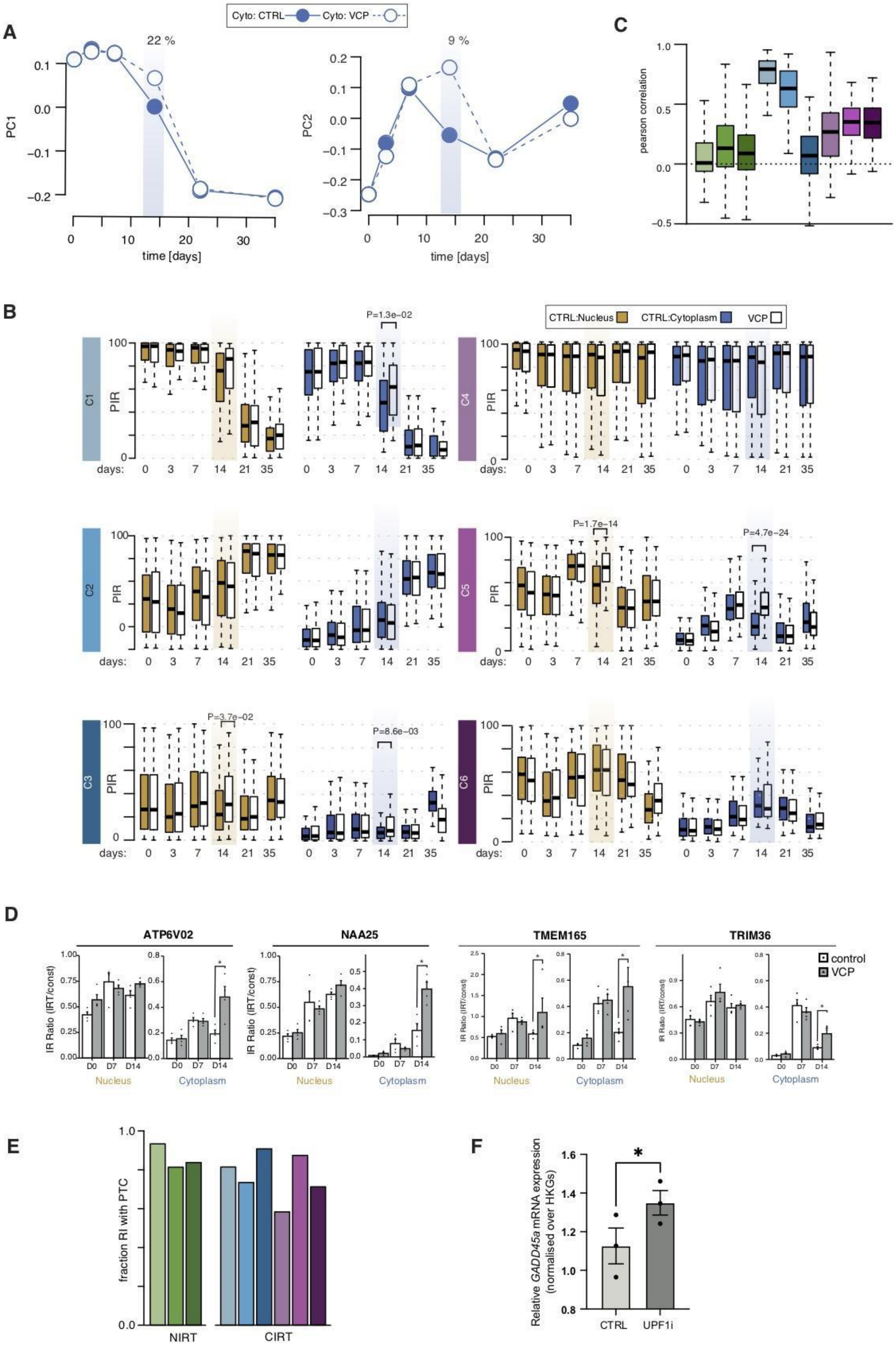

A
Cyto: CTRL
Cyto: VCP
22 %
9 %
PC1
PC2
time [days]
C
pearson correlation
B
CTRL:Nucleus
CTRL:Cytoplasm
VCP
C1
C2
C3
C4
C5
C6
PIR
days:
P=1.3e−02
P=1.7e−14
P=4.7e−24
P=3.7e−02
P=8.6e−03
D
ATP6V02
NAA25
TMEM165
TRIM36
IR Ratio (IRT/const)
control
VCP
D0
D7
D14
Nucleus
Cytoplasm
E
fraction RI with PTC
NIRT
CIRT
F
Relative GADD45a mRNA expression (normalised over HKGs)
CTRL
UPF1i

**Supplemental Figure S3 | A.** Singular value decomposition analysis of the PIR cytoplasmic values of 94,457 introns in n = 48 cytoplasmic samples. Line plots showing the PIR profiles of the first two singular vectors $v_1$and $v_2$, capturing 22% and 9% of the variance in PIR respectively. Filled and empty data points indicate PIR values for the control and VCP$^{mu}$ samples. **B.** Comparison of the distributions of nuclear and cytoplasmic PIR between control (*colored* boxes) and VCP$^{mu}$ (*white* boxes) samples during MN differentiation for the 6 groups of cytoplasmic retained introns. P-values obtained with two-sided Welch *t*-test. Data shown as box plots in which the center line is the median, limits are the interquartile range and whiskers are the minimum and maximum. **C.** Pearson's correlation between nuclear and cytoplasmic PIR for the 9 groups of retained introns. **D.** Bar graphs showing nuclear and cytoplasmic intron retention in C5 candidates, analysed by qPCR, at indicated days during *in vitro* iPSC-derived motor neuron differentiation, in control and VCP$^{mu}$ mutant samples. IR values are calculated as levels of IRT over total transcript expression for each candidate. Data are presented as mean ± S.E.M. Wilcoxon rank sum exact test between controls/mutants, * = P<0.05; not significant where no * is present. Datapoints indicate 4 individual cell lines for both control and mutant. **E.** Fractions of retained introns containing at least one in-frame premature termination codon (PTC) more than 50 nucleotides 5' of the last exon splice junction. **F.** Bar graphs showing qPCR analysis of relative *GADD45a* mRNA expression levels in MNs (DIV=22) after being transfected with siRNA targeting UPF1 (UPF1i) or a scrambled control (CTRL). *GADD45a* is a known target of the NMD pathway. Values are normalized over the geometric mean of housekeeping genes (*GAPDH*, *POLR2B*, *UBE2D3*) and expressed as fold change over mock transfected cells. Data are presented as mean ±S.E.M. Data is derived from three individual cell lines, one experimental block. Paired *t*-test * = p<0.05.

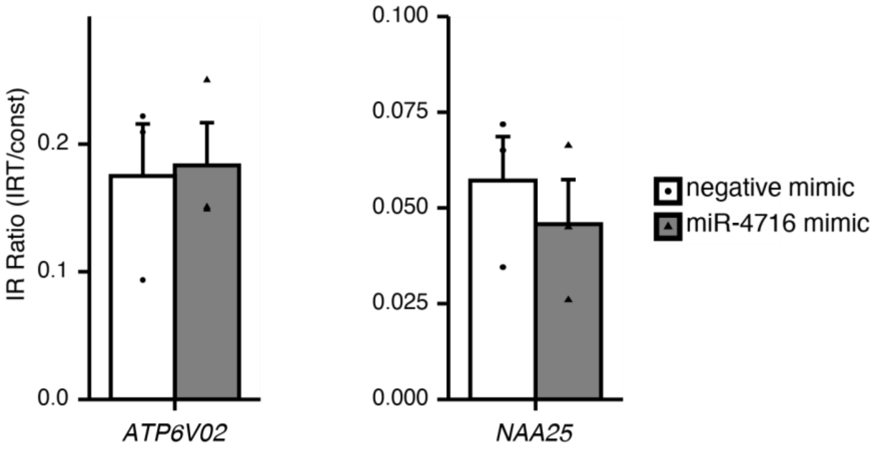


**Supplemental Figure S4 |** Analysis of IR of C5 candidates ATP6V02 and NAA25 in pMN cells (DIV=18) transfected with mimics for miR-4716-5p (miR-4716-5p mimic) or a scrambled negative control (negative mimic). Values are calculated as levels of IRT over total transcript expression for each candidate. Data are presented as mean ± S.E.M. *t*-test * = $p<0.05$; datapoints indicate individual cell lines (n=3).

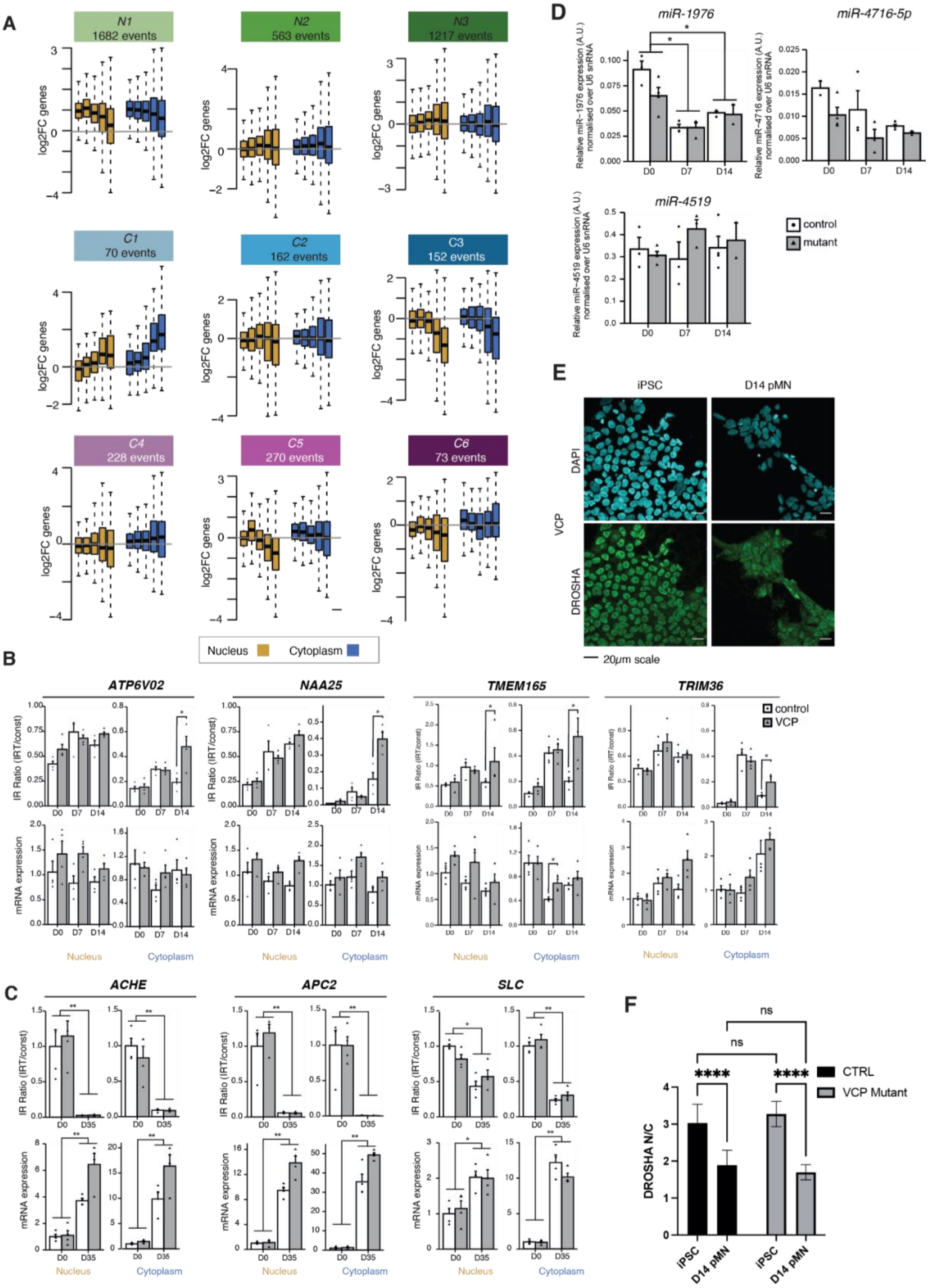
A
N1 1682 events
N2 563 events
N3 1217 events
C1 70 events
C2 162 events
C3 152 events
C4 228 events
C5 270 events
C6 73 events
log2FC genes
Nucleus
Cytoplasm
B
ATP6V02
NAA25
TMEM165
TRIM36
IR Ratio (IRT/const)
mRNA expression
D0 D7 D14
control
VCP
C
ACHE
APC2
SLC
D0 D35
D
miR-1976
miR-4716-5p
miR-4519
Relative miR-1976 expression (A.U.) normalised over U6 snRNA
Relative miR-4716 expression (A.U.) normalised over U6 snRNA
Relative miR-4519 expression (A.U.) normalised over U6 snRNA
control
mutant
E
iPSC
D14 pMN
DAPI
VCP
DROSHA
20μm scale
F
ns
****
CTRL
VCP Mutant
DROSHA N/C
iPSC
D14 pMN

**Supplemental Figure S5 | A.** Changes in gene expression from the RNA-seq data over time in the nucleus (*gold* boxes) and cytoplasm (*blue* boxes) for groups of genes containing the 9 different categories of retained introns. Fold-changes obtained by comparing the $\log_2$ expression level at time of interest ($d_i$ = {3,7,14,22,35}) with the expression level at iPSC stage ($d_0$). Data shown as box plots in which the center line is the median, limits are the interquartile range and whiskers are the minimum and maximum. **B.** qPCR validation of A; bar graphs showing nuclear and cytoplasmic intron retention (*upper*) and relative nuclear and cytoplasmic gene expression levels (*lower*) in C5 candidates, at indicated days during *in vitro* iPSC-derived motor neuron differentiation, in control and $VCP^{mu}$ mutant samples. IR values are calculated as levels of IRT over total transcript expression for each candidate. Gene expression values are normalized over the geometric mean of compartment specific housekeeping genes NIT1 and NFX1 and data are expressed as fold change over CTRL (DIV=0). Data are presented as mean ± S.E.M. Wilcoxon signed rank exact test for gene expression changes between timepoints, Wilcoxon rank sum exact test between controls/mutants, * = P<0.05; not significant where no * is present. Datapoints indicate 4 individual cell lines for both control and mutant, one experimental repeat. **C.** Same as (B) for C1 candidates. Wilcoxon signed rank exact test between timepoints, * = P<0.05. Datapoints indicate 4 individual cell lines for both control and mutant, one experimental repeat. **D.** Bar plots depicting qPCR analysis of miR-1976 (*left*), miR-4716-5p (*right*) and miR-4519 (*lower*) miRNA expression in iPSCs (DIV=0), NPCs (DIV=7) and pMNs (DIV=14). Values are normalized over U6 snRNA. Data are presented as mean ± S.E.M. Wilcoxon signed rank exact test between timepoints, Wilcoxon rank sum exact test between controls/mutants, * = p<0.05; not significant where no * is present. Datapoints indicate 4 individual cell lines for control and mutant, one experimental repeat. **E.** Representative images of DROSHA (green) and DAPI (blue) in iPSCs and pMNs (DIV=14) timepoints of $VCP^{mu}$ cells. **F.** Nuclear to cytoplasmic ratio of mean DROSHA intensity is plotted for iPSC and pMN (DIV=14) cell stages. Two-way ANOVA. ns - non-significant, * =

p<0.05, ** = p<0.01, *** = p<0.001, **** = p<0.0001. Data is derived from n=5 control and n=4 mutant cell lines, one experimental repeat.

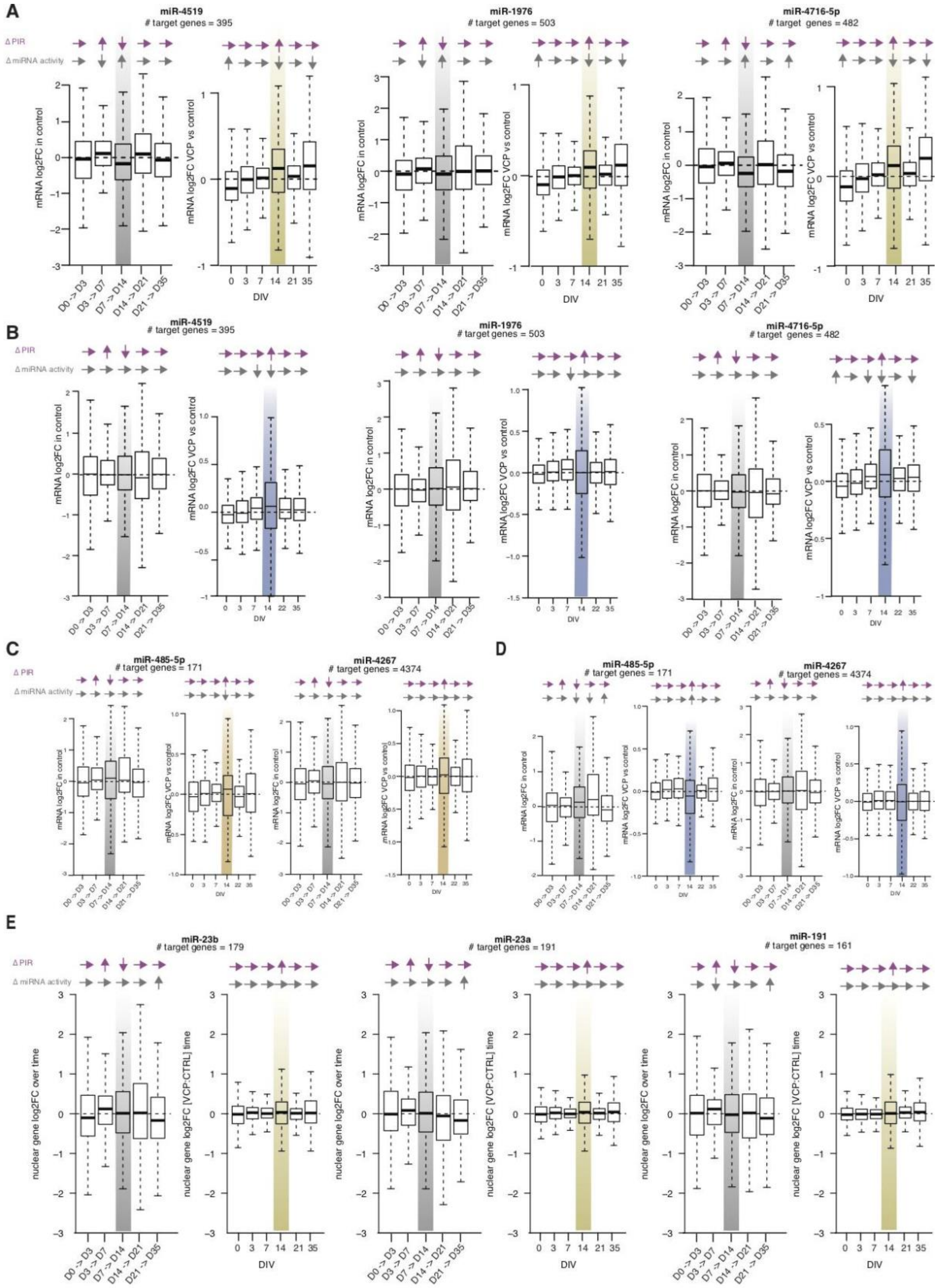

**Supplemental Figure S6 |** Distributions of the changes in expression over time of the control samples (*left*) and between VCP$^{mu}$ and control samples at each time-point (*right*) for the predicted target genes of miR-4519, miR-1976 and miR-4716-5p (Nuclear (**A**), cytoplasmic (**B**)), miR-485-5p and miR-4267 (Nuclear (**C**), cytoplasmic (**D**)), and miR-23b, miR-23a and miR-191 (Nuclear (**E**)). Shaded area = the time-point where the largest changes in cytoplasmic IR are observed. Fold-changes over time obtained by comparing the $\log_2$ expression level at the time of interest ($d_t = \{0, 3,7,14,22,35\}$) with the expression level at previous stage ($d_{t-1}$). Magenta and grey arrows = direction of change of the PIR and predicted miRNA activity respectively, either over time or between VCP$^{mu}$ and control samples.

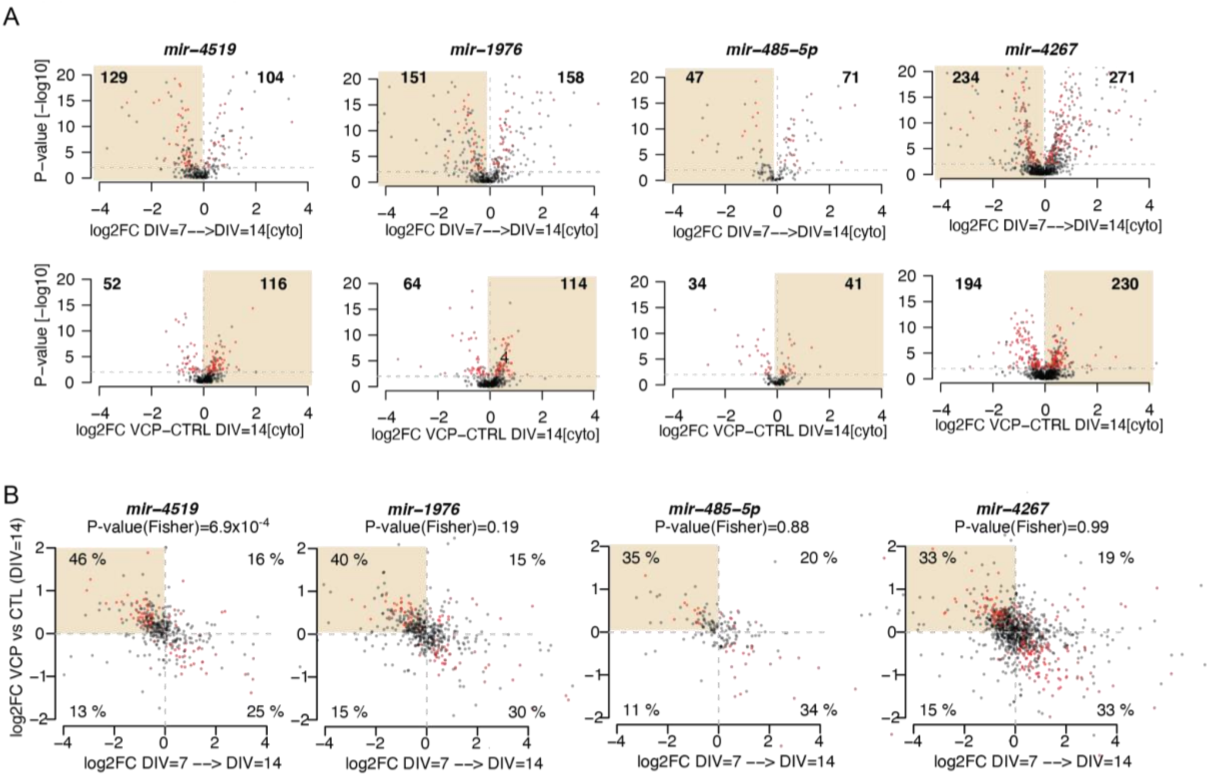


**Supplemental Figure S7 | A.** Volcano plots representing the effect size (i.e., log$_2$(fold-change) on the x-axis) against the statistical significance (i.e., -log$_{10}$(P-Value) on the y-axis) of the changes in the nucleus in gene expression between two consecutive time-points from DIV=7 to DIV=14 for control cell lines (*upper row*) and between control and VCP mutant cell lines at DIV=14 time-point (*lower row*) for the TargetScan predicted target genes for miR-4519, miR-1976, miR-485-5p and miR-4267. Shaded area indicates genes for which the effect size correlates with changes in PIR levels. The number of genes exhibiting significant (P-value <0.05) negative and positive differences in effect size are indicated in the corresponding area. **B.** Scatter plots showing the relationships between the expression changes between DIV=7 and DIV=14 in nuclear control samples (X-axis; log$_2$FC) and between control and VCP cell lines at DIV=14 (Y-axis; log$_2$FC) of the predicted target genes. Red colour indicates genes with P-value <0.05 in both comparisons. P-value obtained from one-side Fisher's exact test comparing the number of genes exhibiting log$_2$FC(DIV=7→DIV=14)<0 and log$_2$FC(VCP vs CTRL at DIV=14) in the pool of miRNA predicted targets with those from the total set of genes.

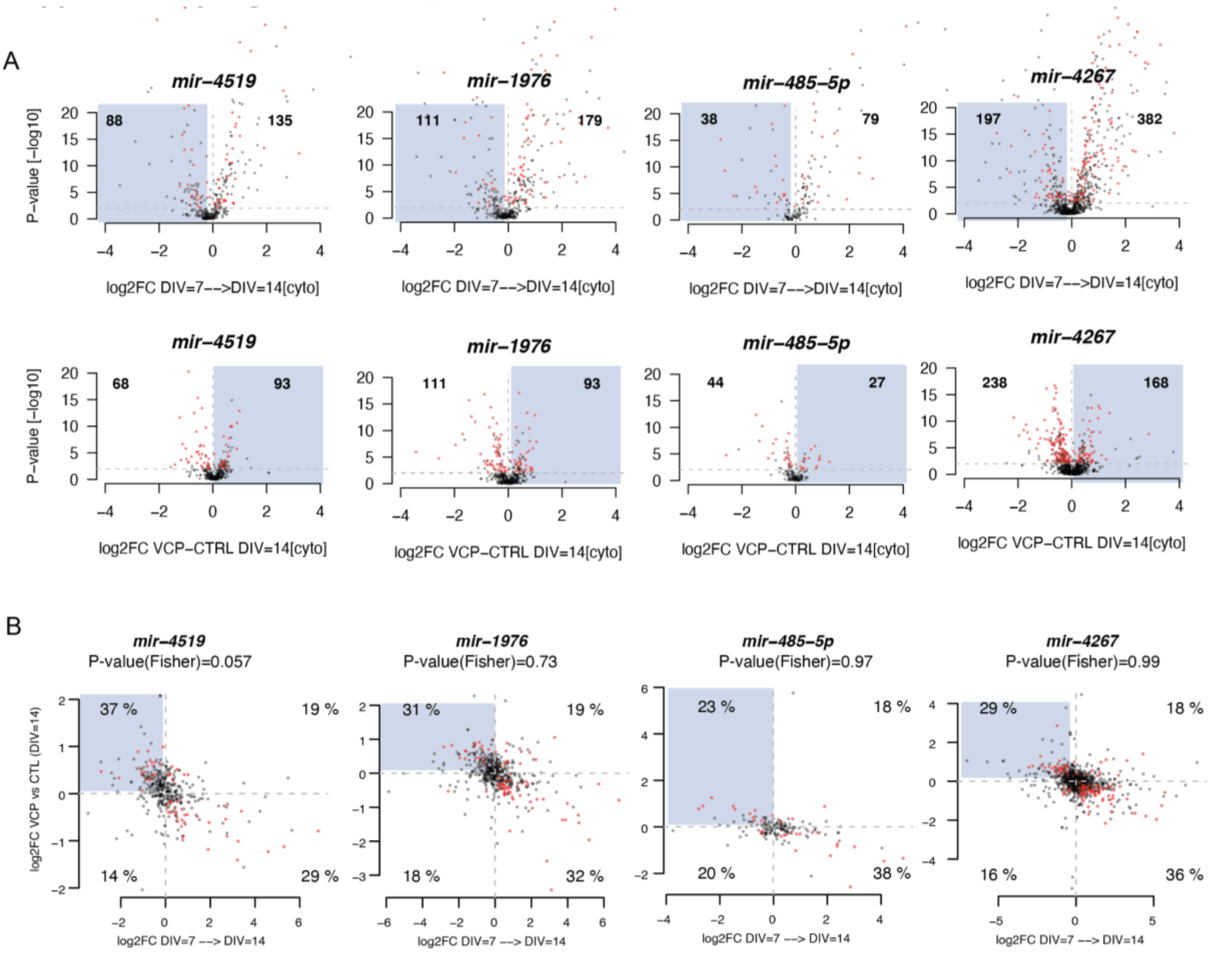


**Supplemental Figure S8 | A.** Volcano plots representing the effect size (i.e., $\log_2$(fold-change) on the x-axis) against the statistical significance (i.e., -$\log_{10}$(P-Value) on the y-axis) of the changes in the cytoplasm in gene expression between two consecutive time-points from DIV=7 to DIV=14 for control cell lines (*upper row*) and between control and VCP mutant cell lines at at DIV=14 time-point (*lower row*) for the TargetScan predicted target genes for miR-4519, miR-1976, miR-485-5p and miR-4267. Shaded area indicates genes for which the effect size correlates with changes in PIR levels. The number of genes exhibiting significant (P-value <0.05) negative and positive differences in effect size are indicated in the corresponding area. **B.** Scatter plots showing the relationships between the expression changes between DIV=7 and DIV=14 in cytoplasmic control samples (X-axis; $\log_2$FC) and between control and VCP cell lines at DIV=14 (Y-axis; $\log_2$FC) of the predicted target genes. Red colour indicates genes with P-value <0.05 in both comparisons. P-value obtained from one-side Fisher's exact test comparing the number of genes exhibiting $\log_2$FC(DIV=7→DIV=14)<0 and $\log_2$FC(VCP vs CTRL at DIV=14) in the pool of miRNA predicted targets with those from the total set of genes.

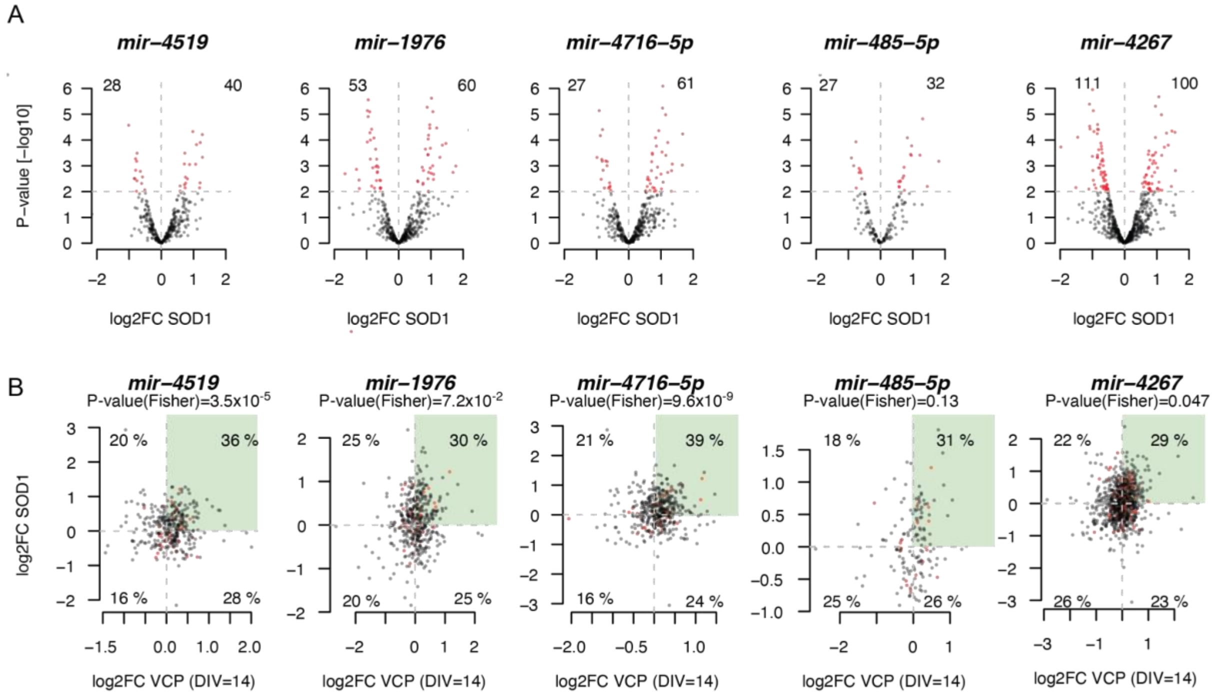


**Supplemental Figure S9 | A.** Volcano plots representing the effect size (i.e., $log_2$(fold-change) on the x-axis) against the statistical significance (i.e., -$log_{10}$(P-Value) on the y-axis) of the changes between control MNs and SOD1$^{mu}$ MNs samples (Kiskinis et al. 2014b) for the TargetScan predicted target genes of miR-4519, miR-1976, miR-4716-5p, miR-485-5p and miR-4267. Shaded area indicates genes for which the effect size correlates with changes in PIR levels. The number of genes exhibiting significant (P-value <0.05) negative and positive differences in effect size are indicated in the corresponding area. **B.** Scatter plots showing the relationships between the expression changes between control and VCP cell lines at DIV=14 (X-axis; $log_2$FC) and between control MNs and SOD1$^{mu}$ MNs samples (Kiskinis et al. 2014b) (Y-axis; $log_2$FC) of the predicted target genes for miR-4519, miR-1976, miR-4716-5p, miR-485-5p and miR-4267. Red colour indicates genes with P-value <0.05 in both comparisons. P-value obtained from one-side Fisher's exact test comparing the number of genes exhibiting $log_2$FC(SOD1 vs CTRL)>0 and $log_2$FC(VCP vs CTRL at DIV=14)>0 in the pool of miRNA predicted targets with those from the total set of genes. ***See also Figure 5.***

## REFERENCES


1. Yeo,G. and Burge,C.B. (2004) Maximum entropy modeling of short sequence motifs with applications to RNA splicing signals. *J. Comput. Biol.*, **11**, 377–394.

2. Moyer,D.C., Larue,G.E., Hershberger,C.E., Roy,S.W. and Padgett,R.A. (2020) Comprehensive database and evolutionary dynamics of U12-type introns. *Nucleic Acids Res.*, **48**, 7066–7078.
